# Morphological and ecological convergence at the lower size limit for vertebrates highlighted by five new miniaturised microhylid frog species from three different Madagascan genera

**DOI:** 10.1371/journal.pone.0213314

**Published:** 2019-03-27

**Authors:** Mark D. Scherz, Carl R. Hutter, Andolalao Rakotoarison, Jana C. Riemann, Mark-Oliver Rödel, Serge H. Ndriantsoa, Julian Glos, Sam Hyde Roberts, Angelica Crottini, Miguel Vences, Frank Glaw

**Affiliations:** 1 Sektion Herpetologie, Zoologische Staatssammlung München (ZSM-SNSB), München, Germany; 2 Division of Evolutionary Biology, Zoologisches Institut, Technische Universität Braunschweig, Braunschweig, Germany; 3 Systematische Zoologie, Department Biologie II, Biozentrum, Ludwig-Maximilians-Universität München, Planegg-Martinsried, Germany; 4 Biodiversity Institute and Department of Ecology and Evolutionary Biology, University of Kansas, Lawrence, KS, United States of America; 5 Mention Zoologie et Biodiversité Animale, Université d’Antananarivo, Antananarivo, Madagascar; 6 Institute of Zoology, Universität Hamburg, Hamburg, Germany; 7 Museum für Naturkunde–Leibniz Institute for Evolution and Biodiversity Science, Berlin, Germany; 8 SEED Madagascar, London, United Kingdom; 9 Oxford Brookes University, Oxford, United Kingdom; 10 CIBIO, Research Centre in Biodiversity and Genetic Resources, InBIO, Campus Agrário de Vairão, Universidade do Porto, Vairão, Portugal; University of Sydney, AUSTRALIA

## Abstract

Miniaturised frogs form a fascinating but poorly understood amphibian ecomorph and have been exceptionally prone to taxonomic underestimation. The subfamily Cophylinae (family Microhylidae), endemic to Madagascar, has a particularly large diversity of miniaturised species which have historically been attributed to the single genus *Stumpffia* largely based on their small size. Recent phylogenetic work has revealed that several independent lineages of cophyline microhylids evolved towards highly miniaturised body sizes, achieving adult snout–vent lengths under 16 mm. Here, we describe five new species belonging to three clades that independently miniaturised and that are all genetically highly divergent from their relatives: (i) a new genus (*Mini* gen. nov.) with three new species from southern Madagascar, (ii) one species of *Rhombophryne*, and (iii) one species of *Anodonthyla*. *Mini mum* sp. nov. from Manombo in eastern Madagascar is one of the smallest frogs in the world, reaching an adult body size of 9.7 mm in males and 11.3 mm in females. *Mini scule* sp. nov. from Sainte Luce in southeastern Madagascar is slightly larger and has maxillary teeth. *Mini ature* sp. nov. from Andohahela in southeast Madagascar is larger than its congeners but is similar in build. *Rhombophryne proportionalis* sp. nov. from Tsaratanana in northern Madagascar is unique among Madagascar’s miniaturised frogs in being a proportional dwarf, exhibiting far less advanced signs of paedomorphism than other species of similar size. *Anodonthyla eximia* sp. nov. from Ranomafana in eastern Madagascar is distinctly smaller than any of its congeners and is secondarily terrestrial, providing evidence that miniaturisation and terrestriality may be evolutionarily linked. The evolution of body size in Madagascar’s microhylids has been more dynamic than previously understood, and future studies will hopefully shed light on the interplay between ecology and evolution of these remarkably diverse frogs.

## Introduction

Miniaturisation is a common phenomenon in amphibians [1–4], and is especially widespread and extreme in frogs [4–6]. A large proportion of the world’s smallest frogs belong to the highly diverse family Microhylidae [5–8]. Microhylid frogs exhibit high degrees of osteological variation, especially in the morphology of elements of the skull, hands, feet, and pectoral girdle [9]. The smallest species among the microhylids do not belong to a single clade however, but rather occur in various different subfamilies spread across the tropics, including New Guinea [5, 6, 8], Borneo [10, 11], South America [12, 13], and Madagascar [14, 15]. Even within single microhylid subfamilies, multiple independent instances of miniaturisation are evident from the interdigitation of miniaturised and non-miniaturised species in the respective phylogenetic trees [8, 15]. One of the best examples is the subfamily Cophylinae, endemic to Madagascar [15].

**Fig 1:**
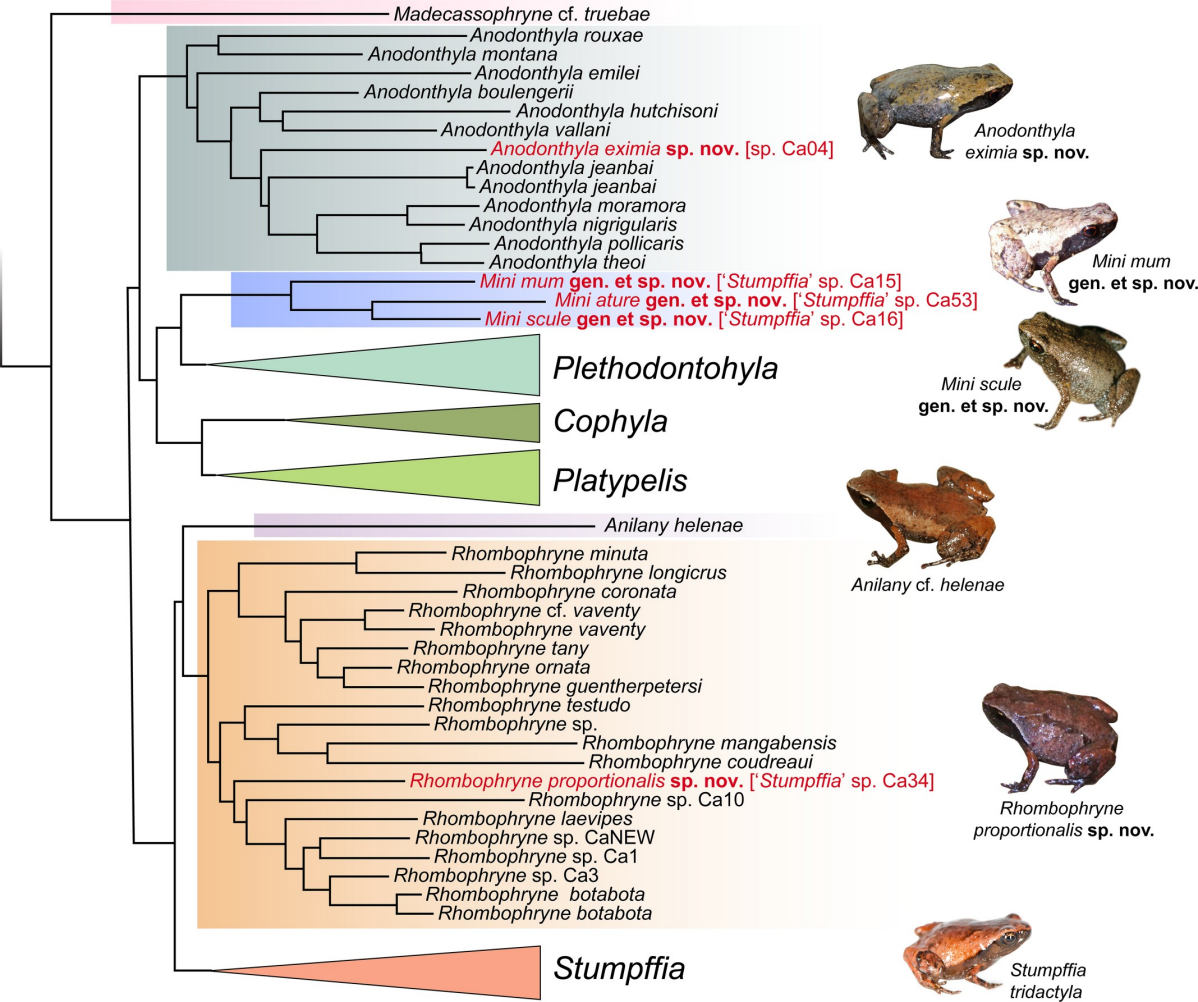
Stylised representation of the latest phylogeny of the microhylid subfamily Cophylinae. Based on the phylogenetic tree of Tu et al. [20] (reconstructed from a concatenated multi-gene DNA sequence data set) with numerous nomenclatural updates. See that paper for support values and phylogenetic context. The sister species of *Rhombophryne proportionalis* sp. nov. is an undescribed candidate species (*R*. sp. Ca7) that is not included in this phylogeny; its taxonomy will be treated elsewhere. Taxa highlighted in red are those described herein. Inset photos show a selection of the new species described herein, and representatives of the other small-bodied genera, *Stumpffia* and *Anilany*. Inset photos are not to scale.

Cophylinae currently consists of 103 described species, divided across eight recognised genera. Two of the largest microhylids, *Platypelis grandis* and *Plethodontohyla inguinalis* are members of this subfamily, but it also contains frogs that are among the smallest in the world, such as *Stumpffia contumelia* (adult snout–vent length [SVL] 8.0–8.9 mm [14]). Historically all small to miniaturised terrestrial cophylines were placed in the genus *Stumpffia*, as they are superficially homogeneous in external morphology, bioacoustics, and ecology, but even early molecular phylogenetic results suggested that their diversity exceeded a single genus [16, 17]. Several lineages of cophylines have independently miniaturised, converging on a diminutive phenotype, and belonging to a number of different genus-level clades which, however, have not yet all been taxonomically named. The latest phylogenetic reconstructions [15, 18–21] have clarified this picture; Fig 1 illustrates a simplified view of the latest phylogeny of Tu et al. [20], with emphasis on previously undescribed, miniaturised lineages that do not belong to *Stumpffia*.

In 2016, we highlighted three deep clades of miniaturised frogs within the Cophylinae that we considered probably distinct from *Stumpffia* [15]. These were: (i) ‘*S*.’ *helenae*, which we transferred to a new genus *Anilany* in order to preserve the monophyly of *Stumpffia*—this decision was challenged by Peloso et al. [22], who preferred to subsume *Stumpffia*, *Anilany*, and the generally much larger and morphologically strongly distinct *Rhombophryne* into a single genus, but evidence from morphology and genetics argues against this lumping approach [23]; (ii) a clade of two undescribed lineages from south-eastern Madagascar (provisionally named ‘*S*.’ spp. Ca15 and Ca16), originally identified by Wollenberg et al. [17], which consistently fall sister to the large-bodied *Plethodontohyla*—a third member of this clade, ‘*S*.’ sp. Ca53 was sequenced and deposited in GenBank by Rakotoarison et al. [14] and included in the phylogeny of Tu et al. [20]; and (iii) two frogs from Tsaratanana (*Rhombophryne* sp. Ca7 and ‘*S*.’ sp. Ca34), which were topologically unstable—these have since been found to fall within *Rhombophryne* ([20]; unpublished data), and only the latter is miniaturised, while the former is a frog of moderate-size (SVL 23.5–26.5 mm).

The phylogenetic position of *Stumpffia tridactyla* was also unstable [15, 22, 23] and the species was flagged as potentially divergent [23]. In light of our recent revision of the entirety of *Stumpffia* [14] and osteological data (unpublished observations), this species and its sister species, *S*. *contumelia*, form a clade sister to all other *Stumpffia* that we do not, at this time, consider to warrant genus-level recognition.

In previous work, we overlooked another miniaturised species that was included in our phylogeny [15, 20, 24] but not morphologically examined, namely *Anodonthyla* sp. Ca04 Ranomafana (ZCMV 204). The only available specimen of this species measures 11.3 mm SVL, and genetically and morphologically can be ascribed to *Anodonthyla*, representing an additional miniaturisation event within the Cophylinae.

In summary, there are three miniaturised species within an undescribed genus-level clade sister to *Plethodontohyla*, one miniaturised species of *Rhombophryne*, and one miniaturised species of *Anodonthyla*, all of which are awaiting taxonomic description. In the present paper, we provide formal descriptions of these taxa, with the aim of facilitating future research on the convoluted evolution of the frogs of the subfamily Cophylinae.

## Materials and methods

### Ethics statement

Approval for this study by an Institutional Animal Care and Use Committee (IACUC) was not required by Malagasy law, but all work complied with the guidelines for field research compiled by the American Society of Ichthyologists and Herpetologists (ASIH), the Herpetologists’ League (HL), and the Society for the Study of Amphibians and Reptiles (SSAR). All field research, collecting of specimens, including in situ euthanasia of specimens, were approved by the Madagascan Ministère de l’Environnement des Eaux et des Forêts (Direction des Eaux et Forêts, DEF) under the following permits:

64/10/MEF/SG/DGF/DCB.SAP/SLRSE, 238-MINENV.EF/SG/DGEF/DPB/SCBLF/RECH, 045/12/MEF/SG/DGF/DCB.SAP/SCB, 238-MINENVEF/SG/DGEF/DPB/SCBLF, 218-MEEF/DEF/SPN/FFE/AUT, 198/16/MEEF/SG/DGF/DSAP/SCB.Re. Export of specimens was approved by the DEF under permits: 135N-EA07/MG10, 103C-EA03/ MG05, 044N-EA04/MG12, 094C-EA03/MG04, 094C-EA03/MG04, export 356N-EA12/MG16. Specimens were anaesthetised and subsequently euthanized following approved methods (MS222 solution; approved by the American Veterinary Medical Association) that do not require approval by an ethics committee, after consultation of the Animal Welfare Officer of TU Braunschweig.

### Voucher specimens

Our study areas were as follows: Sainte Luce (ca. 24.75–24.76°S, 47.17–47.18°E); Tsaratanana (ca. 14.08–14.12°S, 48.97–48.99°E); Manombo (ca. 23.02–23.03°S, 47.72–47.73°E); Nahampoana (ca. 24.98°S, 46.98°E); Andohahela (ca. 24.75°S, 46.85°E). Field numbers used in this study refer to the zoological collections of Frank Glaw (FGZC), Miguel Vences (ZCMV), David R. Vieites (DRV), Serge H. Ndriantsoa (NSH), Angelica Crottini (ACZCV), and Sam Hyde Roberts (SHR). The following institutional acronyms are used: Zoologische Staatssammlung München, Munich (ZSM), amphibian collections of the Mention Zoologie et Biodiversité Animale of the Université d’Antnanarivo (UADBA-A), Museum für Naturkunde, Berlin (ZMB), Muséum National d’Histoire Naturelle, Paris (MNHN), Zoologische Forschungsmuseum Alexander Koenig, Bonn (ZFMK), Zoological Museum of Amsterdam (ZMA)—today part of the Naturalis Biodiversity Centre, Leiden. Collected voucher specimens were fixed in the field in 90% ethanol and some subsequently transferred to 70% ethanol for long-term storage. Tissue samples (muscle or a limb) were preserved in 99% ethanol in the field, before specimen fixation.

### Morphological measurements

Morphological examination was done under a binocular dissecting microscope. Measurements were taken with a digital calliper to the nearest 0.01 mm by MDS and rounded to the nearest 0.1 mm, with ratios calculated prior to rounding to avoid compound rounding errors. Measurement scheme followed our previous work [25–28]. The scheme is repeated here for convenience verbatim from Scherz et al. [25]: ‘SVL (snout–vent length), HW (maximum head width), HL (head length, from the maxillary commissure to the snout tip. Note: this is measured along the jaw, and not parallel to the longitudinal axis of the animal), ED (horizontal eye diameter), END (eye–nostril distance, from the anterior eye to the posterior of the naris), NSD (nostril–snout tip distance, from the centre of the naris), NND (internarial distance, from the centre of each naris), TDH (horizontal tympanum diameter), TDV (vertical tympanum diameter), HAL (hand length, from the carpal–radioulna articulation to the tip of the longest finger), LAL (lower arm length, from the carpal–radioulna articulation to the radioulna–humeral articulation), UAL (upper arm length, from the radioulna–humeral articulation to the trunk, measured along the posterior aspect of the arm), FORL (forelimb length, given by the sum of HAL, LAL, and UAL), FOL (foot length, from the tarsal–metatarsal articulation to the tip of the longest toe), TARL (tarsal length, from the tarsal–metatarsal articulation to the tarsal–tibiofibular articulation), FOTL (foot length including tarsus, given by the sum of FOL and TARL), TIBL (tibiofibula length, from the femoral–tibiofibular articulation to the tarsal–tibiofibular articulation), TIBW (maximum tibiofibula [= shank] width), THIL (thigh length, from the vent to the femoral–tibiofibular articulation), THIW (thigh width at thickest point, measured in supine position), HIL (hindlimb length, given by the sum FOL, TARL, TIBL, and THIL), IMCL (maximum length of inner metacarpal tubercle), IMTL (maximum length of the inner metatarsal tubercle).’ Comparison to *Stumpffia* species based on external morphology is done in relation to the data presented by Rakotoarison et al. [14]. Morphological terminology referring to digits of the hands and feet is based on Rakotoarison et al. [14].

### Micro-Computed Tomography

Micro-Computed Tomography (micro-CT) scans were produced in a nanotom m cone-beam scanner (phoenix|x, GE Measurement & Control, Wunstorf, Germany). Specimens were placed in small vessels such as Eppendorf tubes or film canisters and fixed in place using polystyrene and thin wooden struts. A small volume of ethanol was added to the container to prevent desiccation, and a lid was firmly shut to prevent excessive evaporation. Scan times were maximally 30 minutes. Standard scanning parameters were 750 ms exposures at 140 kV and 80 μA for 2440 projections under fastscan parameters, with a 0.1 mm copper filter and a tungsten target, but small deviations were necessary in some cases. Scans were initially reconstructed in datos|x reconstruct software (GE Measurement & Control) and were then visualised in 8-bit under phong volume rendering settings in VG Studio Max 2.2 (Volume Graphics GMBH, Heidelberg, Germany) using a custom preset (available upon request). Osteological description was based on volume-renderings of the micro-CT data, following recommendations of Scherz et al. [25]. Osteological terminology follows Trueb [29–31] and Fabrezi and Alberch [32]. Rotational videos of micro-CT data were produced in VG Studio Max 2.2 following the methods outlined in Scherz et al. [25], and these together with DICOM image stacks of the scans produced in this study are available from http://morphosource.org/Detail/ProjectDetail/Show/project_id/464.

### Bioacoustics

Recordings were made in the field using digital recorders (Tascam DR07 or DR05, Tascam DR-40, Roland EDIROL R-09) with internal or external (Sennheiser ME66/K6) microphone, or with an analogue Sony WM-D6C tape recorder with a Vivanco EM 238 external microphone in the case of the new *Anodonthyla* and early recordings from Nahampoana. Call analysis was conducted in Audacity 2.2.0 (https://github.com/audacity/audacity). Recordings with background wind were run through a decaying hi-pass filter in Audacity set at ca. 3 kHz to clean the audio prior to analysis. Frequency information was obtained through Fast Fourier Transformation (FFT; width 1024 points). The spectrogram was obtained using the Hanning window function with 256 bands resolution. Bioacoustic parameters are given as mean ± standard deviation, with range in brackets. We measured dominant frequency, call duration, inter-call silent interval, and, where relevant, notes per call. Terminology follows the note-centred approach of Köhler et al. [33].

### Molecular phylogenetics

We here present only minimal genetic data, because relevant genetic conclusions have been reported elsewhere [15, 17–21]; see Fig 1. Sequences of a 3’ segment of the *16S rRNA* mtDNA gene were downloaded from GenBank (see S1 Table for full list of GenBank accession numbers used) and complemented by sequences of 11 additional specimens to verify their identity (GenBank accession numbers of new sequences: MK459307–MK459317). Separate alignments were constructed for the three genera concerned here: (1) *Mini* gen. nov. and *Plethodontohyla*, (2) *Rhombophryne*, and (3) *Anodonthyla*. Sequences were aligned using MUSCLE [34], and manually checked and trimmed in MEGA7 [35]. We herein also report uncorrected pair-wise distances (p-distances) between species in the *16S rRNA* gene, calculated in MEGA7 [35].

### Taxonomic approach

Species described herein were identified initially based on molecular differentiation from other species, and morphological characters were then interrogated for diagnostic features, taking into account their phylogenetic position based on molecular data. Description schemes loosely follow Rakotoarison et al. [14], especially in the respect of comparing new material with a minimum possible set of other species in order to promote brevity. We follow the recommendations of Vences et al. [36] with regard to the economy of change in our consideration of the taxonomy of higher taxa.

### Nomenclatural acts

The electronic edition of this article conforms to the requirements of the amended International Code of Zoological Nomenclature (ICZN), and hence the new names contained herein are available under that Code from the electronic edition of this article. This published work and the nomenclatural acts it contains have been registered in ZooBank, the online registration system for the ICZN. The ZooBank LSIDs (Life Science Identifiers) can be resolved and the associated information viewed through any standard web browser by appending the LSID to the prefix ‘http://zoobank.org/‘. The LSID for this publication is: urn:lsid:zoobank.org:pub:91E597C8-7A80-46F9-B0E8-61F2524400F7. The journal’s eISSN is 1932–6203. The article has been archived and is available from the following repositories: PubMed Central and LOCKSS.

### Miniaturisation terminology

Clarke [4] discussed the definition of miniaturisation in the different clades of amphibians. He considered 25–30 mm SVL to represent the ‘critical division in anurans,’ with specimens of smaller sizes ‘exhibiting physiological and ecological modifications’ associated with miniaturisation. Thus, he considered 20–25 mm specimens ‘small’, and specimens below 20 mm ‘miniaturised’, with no further refinement for smaller taxa. Trueb and Alberch [37] considered any frog smaller than 25 mm to be ‘small’, with the further categorisation of ‘dwarf’ for frogs under 14 mm. Clarke [4] considered this scheme to be ‘too restrictive in the case of the extreme-small-size category’. We here opt for a compromise between these two schemes and consider species below 12 mm to be ‘extremely miniaturised’, below 16 mm to be ‘highly miniaturised’, below 20 mm to be ‘miniaturised’, and below 24 mm to be ‘small’.

#### Results

The miniaturised members of the subfamily Cophylinae, considering the described diversity and the undescribed lineages outlined in the Introduction, are morphologically, ecologically, and bioacoustically highly similar: they are terrestrial, leaf-litter dwelling frogs that predominantly emit their advertisement calls during the day. Their calls (with few exceptions, including the undescribed miniaturised *Rhombophryne*) consist of single, high-pitched, tonal notes emitted at regular intervals. Most if not all use ‘slow-motion’ walking [38, 39] as their primary mode of locomotion when undisturbed. All exhibit outward signs of reduction in length and/or number of digits, and almost all lack strongly expanded terminal discs (present only in larger species of *Stumpffia*, and in *Anilany helenae*). Eye size is rather small, but nevertheless relatively larger compared to head length (ED up to 57% of HL; Table 1) than in non-miniaturised cophylines (e.g. maximum 45% in the *Rhombophryne serratopalpebrosa* species group; [25]). Finally, they are all inconspicuous in colour dorsally, with only a few larger species of *Stumpffia* possessing red on the hidden surfaces of their legs and on their venters [14].

**Table 1:** Morphological measurements (in mm) of new species of miniaturised cophyline microhylids. Measurement abbreviations are listed in the Materials and Methods. A = Adult, M = Male, F = Female (lowercase indicates probable subadult), p = paratype, h = holotype (holotypes are also bolded), na = not available.

Collection Number	Field Number	Species	Status	Sex	SVL	HW	HL	ED	END	NSD	NND	TDH	TDV	HAL	UAL	LAL	FORL	FARL	THIL	THIW	TIBL	TIBW	TARL	FOL	FOTL	HIL	IMCL	IMTL
**ZSM 1826/2010**	**ZCMV 12404**	***Rhombophryne proportionalis* sp. nov.**	**h**	**M**	**12.3**	**4.1**	**2.4**	**1.2**	**0.6**	**0.7**	**1.5**	**0.7**	**0.7**	**2.4**	**2.1**	**1.9**	**6.4**	**4.3**	**4.2**	**2.7**	**4.2**	**1.8**	**2.3**	**4.4**	**6.7**	**15.0**	**0.4**	**0.5**
ZSM 636/2014	DRV 6224	*Rhombophryne proportionalis* sp. nov.	p	M	11.0	4.1	2.6	1.0	0.5	0.7	1.2	0.8	0.7	1.6	1.6	2.1	5.4	3.8	4.3	2.1	3.9	1.4	2.4	3.9	6.3	14.6	0.3	0.3
ZSM 1840/2010	ZCMV 12405	*Rhombophryne proportionalis* sp. nov.	p	M	11.5	3.8	2.4	1.1	0.6	0.6	1.1	na	na	2.0	1.3	1.8	5.1	3.8	4.4	2.2	3.9	1.3	1.8	3.8	5.6	13.9	0.3	0.5
**ZSM 861/2014**	**ZCMV 14788**	***Mini mum* gen. et sp. nov.**	**h**	**M?**	**8.2**	**3.0**	**2.1**	**1.2**	**0.5**	**0.6**	**1.6**	**0.6**	**0.6**	**1.5**	**1.4**	**1.6**	**4.5**	**3.1**	**3.5**	**1.9**	**4.1**	**1.4**	**2.3**	**3.1**	**5.4**	**13.0**	**0.0**	**0.3**
ZSM 862/2014	ZCMV 14789	*Mini mum* gen. et sp. nov.	p	M?	8.8	3.0	2.2	1.2	0.6	0.7	1.2	0.4	0.4	1.1	1.8	1.6	4.5	2.7	4.6	1.7	4.1	1.3	2.7	3.8	6.4	15.1	0.0	0.4
ZMA 20172	ZCMV 557	*Mini mum* gen. et sp. nov.	p	M?	9.2	3.3	2.3	1.2	0.5	0.7	1.2	0.3	0.4	1.5	1.6	1.9	5.0	3.4	3.6	1.7	4.3	1.4	2.8	3.8	6.6	14.5	0.2	0.4
ZMB 83194	NSH 2583	*Mini mum* gen. et sp. nov.	p	F	11.3	3.5	2.7	1.3	0.6	0.6	1.2	0.6	0.7	1.7	1.8	2.0	5.5	3.7	5.3	1.7	4.6	1.4	2.8	4.1	6.9	16.7	0.0	0.0
ZMB 81993	NSH 2584	*Mini mum* gen. et sp. nov.	p	M	9.7	3.2	2.8	1.1	0.7	0.7	1.2	0.5	0.5	1.3	1.5	1.7	4.6	3.0	4.5	1.6	3.8	1.1	2.5	3.5	5.9	14.2	0.0	0.0
**ZSM 5943/2005**	**FGZC 2662**	***Mini scule* gen. et sp. nov.**	**h**	**M?**	**10.5**	**3.3**	**2.8**	**1.1**	**0.7**	**0.7**	**1.2**	**0.6**	**0.6**	**1.8**	**1.8**	**1.9**	**5.5**	**3.6**	**4.0**	**1.6**	**4.6**	**1.4**	**2.6**	**3.6**	**6.3**	**14.8**	**0.0**	**0.2**
ZSM 5942/2005	FGZC 2661	*Mini scule* gen. et sp. nov.	p	M?	9.9	3.3	2.5	1.2	0.6	0.6	1.1	0.6	0.6	na	1.6	na	na	na	4.2	1.9	4.3	1.2	na	na	na	na	na	na
ZSM 577/2016	ACZCV 0383	*Mini scule* gen. et sp. nov.	p	A	10.8	3.6	2.8	1.4	0.6	0.7	1.4	0.5	0.4	1.7	1.9	2.3	5.9	4.0	4.5	2.0	4.3	1.6	2.6	4.0	6.6	15.4	0.3	0.3
UADBA-A Uncatalogued	ACZCV 0384	*Mini scule* gen. et sp. nov.	p	M	10.2	3.4	2.7	1.2	0.6	0.7	1.3	0.7	0.7	1.8	2.1	2.2	6.0	4.0	4.8	1.9	4.7	1.7	3.1	4.1	7.2	16.6	0.2	0.5
ZSM 578/2016	ACZCV 0385	*Mini scule* gen. et sp. nov.	p	A	10.7	3.5	2.8	1.4	0.6	0.8	1.4	0.7	0.7	1.7	2.0	2.4	6.1	4.1	5.1	1.8	4.6	1.4	3.2	4.1	7.4	17.1	0.2	0.3
UADBA-A Uncatalogued	ACZCV 0386	*Mini scule* gen. et sp. nov.	p	A	9.2	3.2	2.4	1.2	0.7	0.6	1.1	0.5	0.5	1.4	1.5	1.9	4.8	3.3	4.0	1.8	4.2	1.4	2.5	3.4	5.9	14.2	0.3	0.4
UADBA-A Uncatalogued	ACZCV 0387	*Mini scule* gen. et sp. nov.	p	f	8.4	3.1	2.2	1.1	0.6	0.6	1.2	0.6	0.6	1.3	1.4	1.6	4.4	2.9	4.1	1.7	4.0	1.4	2.4	3.7	6.0	14.1	0.3	0.5
ZFMK 53775	—	*Mini* cf. *scule* Nahampoana	—	A	10.5	4.0	2.8	1.4	0.6	0.8	1.4	0.6	0.6	1.8	1.8	2.3	5.9	4.1	4.9	2.0	4.1	1.7	2.7	4.0	6.7	15.7	0.3	0.6
**ZSM 86/2004**	**FGZC 0151**	***Mini ature* gen. et sp. nov.**	**h**	**A**	**14.9**	**5.6**	**3.8**	**1.5**	**0.8**	**0.8**	**1.8**	**0.7**	**0.8**	**2.3**	**1.6**	**2.5**	**6.4**	**4.8**	**4.6**	**2.7**	**5.1**	**2.0**	**2.9**	**5.0**	**7.9**	**17.6**	**0.3**	**0.3**
**ZMA 20246**	**ZCMV 204**	***Anodonthyla eximia* sp. nov.**	**h**	**M**	**11.3**	**3.5**	**2.6**	**1.2**	**0.7**	**0.8**	**1.4**	**0.6**	**0.7**	**1.6**	**1.3**	**1.7**	**4.6**	**3.3**	**4.1**	**1.7**	**4.4**	**1.6**	**2.8**	**3.8**	**6.6**	**15.1**	**0.3**	**0.4**

Despite these strong similarities, detailed morphological (including osteological) assessment of the specimens representing the deep lineages outlined above (in the Introduction) yielded characters that differ significantly between them and all other cophylines. Of particular note are characters of the skull and hands, which show strong signs of miniaturisation and corresponding convergence, but also retain hallmarks of their evolutionary history shared with their non-miniaturised closest relatives.

Specimens belonging to the miniaturised clade sister to *Plethodontohyla* differ from all *Stumpffia* species by their curved clavicles, broad contact between the neopalatine and the straight-edged cultriform process of the parasphenoid (vs narrow contact and obliquely-edged), and a fused or lost carpal 2 (present in *Stumpffia*). The three deep lineages within this clade (Fig 1) differ from one another strongly in the state of the palate and dentition: the lineage from Manombo (‘*Stumpffia*’ sp. Ca15 in Scherz et al. [15]) lacks teeth altogether, while that from Sainte Luce (‘*Stumpffia*’ sp. Ca16 in Scherz et al. [15]) has both maxillary and premaxillary teeth. The lineage from Andohahela (‘*Stumpffia*’ sp. Ca53 in Tu et al. [20]) differs from both of these lineages in having a much larger body size, from the lineage from Manombo by possessing teeth, and from the lineage from Sainte Luce by a number of skull characters, including proportionally smaller braincase, broader contact between quadratojugal and maxilla, and proportionally smaller nasals.

Specimens belonging to the miniaturised *Rhombophryne* species (‘*Stumpffia*’ sp. Ca34 in Scherz et al. [15]) are morphologically (and genetically) homogeneous. This species differs from all other miniaturised species of the subfamily Cophylinae by the presence of vomerine teeth. It also differs from most *Stumpffia* by the total absence of clavicles, the presence of a strong lateral head colour border (thus far known only in *Anilany* and two much larger *Stumpffia* species, *S*. *be* and *S*. *hara* among small-sized cophylines), and very different hand and skull morphology, which is similar to larger *Rhombophryne* in its possession of a distinct first finger and toe (strongly reduced or absent in most *Stumpffia* species, especially those of similar size). Numerous characters separate this species from all other *Rhombophryne* species, the most distinct of which being its considerably smaller body size. It also has a highly distinct call.

The sole available specimen of the miniaturised lineage within the genus *Anodonthyla*, ZMA 20246 (ZCMV 204), is an adult male, collected while presumably calling. It retains the synapomorphies of that genus, some of which are also related to sexual dimorphism in other members of the genus: a long and cultriform prepollex exceeding the length of the first metacarpal (present only in males of *Anodonthyla*), curved clavicles with distal knobs, and T-shaped terminal phalanges of toes 2–5 and the third finger. It differs from all *Anodonthyla* species by the absence of ossification in the sphenethmoid, and by the absence of strongly expressed humeral spurs or crests found in males of many species [24].

In summary, each of the five undescribed, species-level lineages of miniaturised frogs within the Cophylinae possess diagnostic characters. The three species forming a genus-level clade share synapomorphies that distinguish them from their sister genus, *Plethodontohyla*, and indeed all other miniature frogs from Madagascar. We therefore describe a new genus containing three new species, and one new species each of *Rhombophryne* and *Anodonthyla*.

### *Mini* gen. nov.

urn:lsid:zoobank.org:act:67171236-00A2-428B-8194-584BF52E84E6

(Figs 2–9, Table 1)

**Fig 2:**
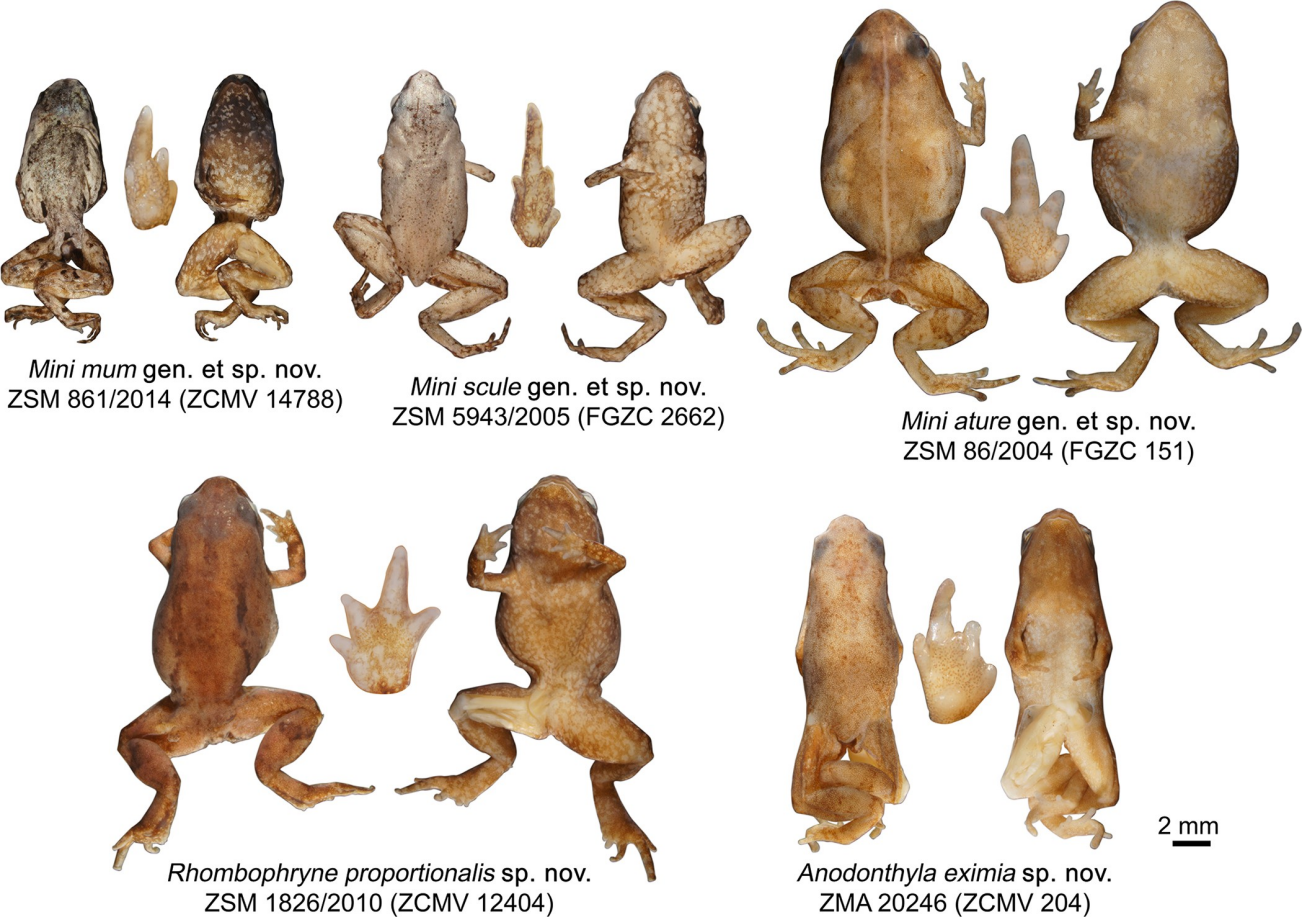
Holotypes of the new species described in this paper and their hands. Whole specimens in dorsal (left) and ventral (right) view. Hand images not to scale.

**Fig 3:**
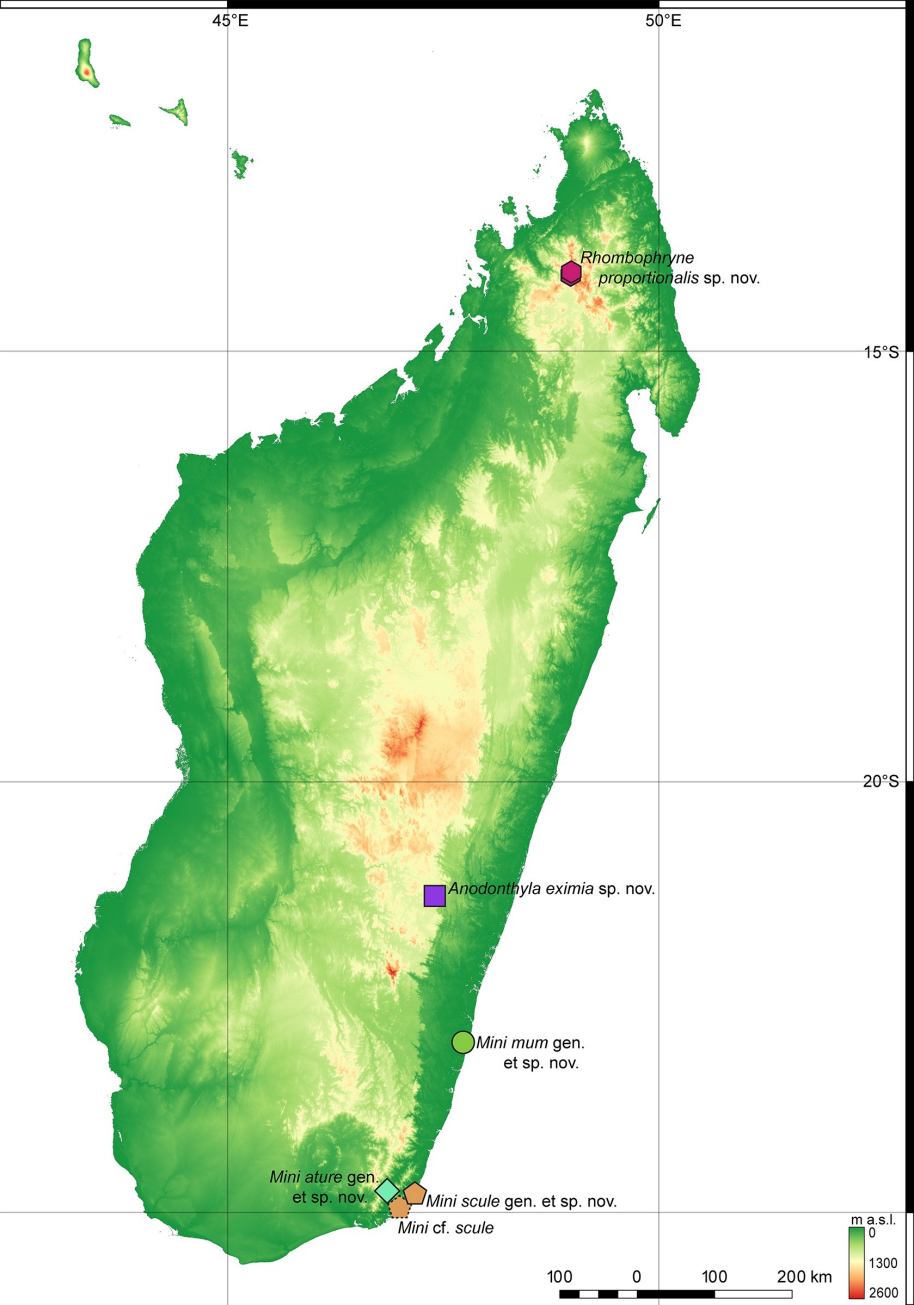
Currently known localities of the new taxa described in this paper. The base map is USGS SRTM 1-Arc second digital elevation model.

**Fig 4:**
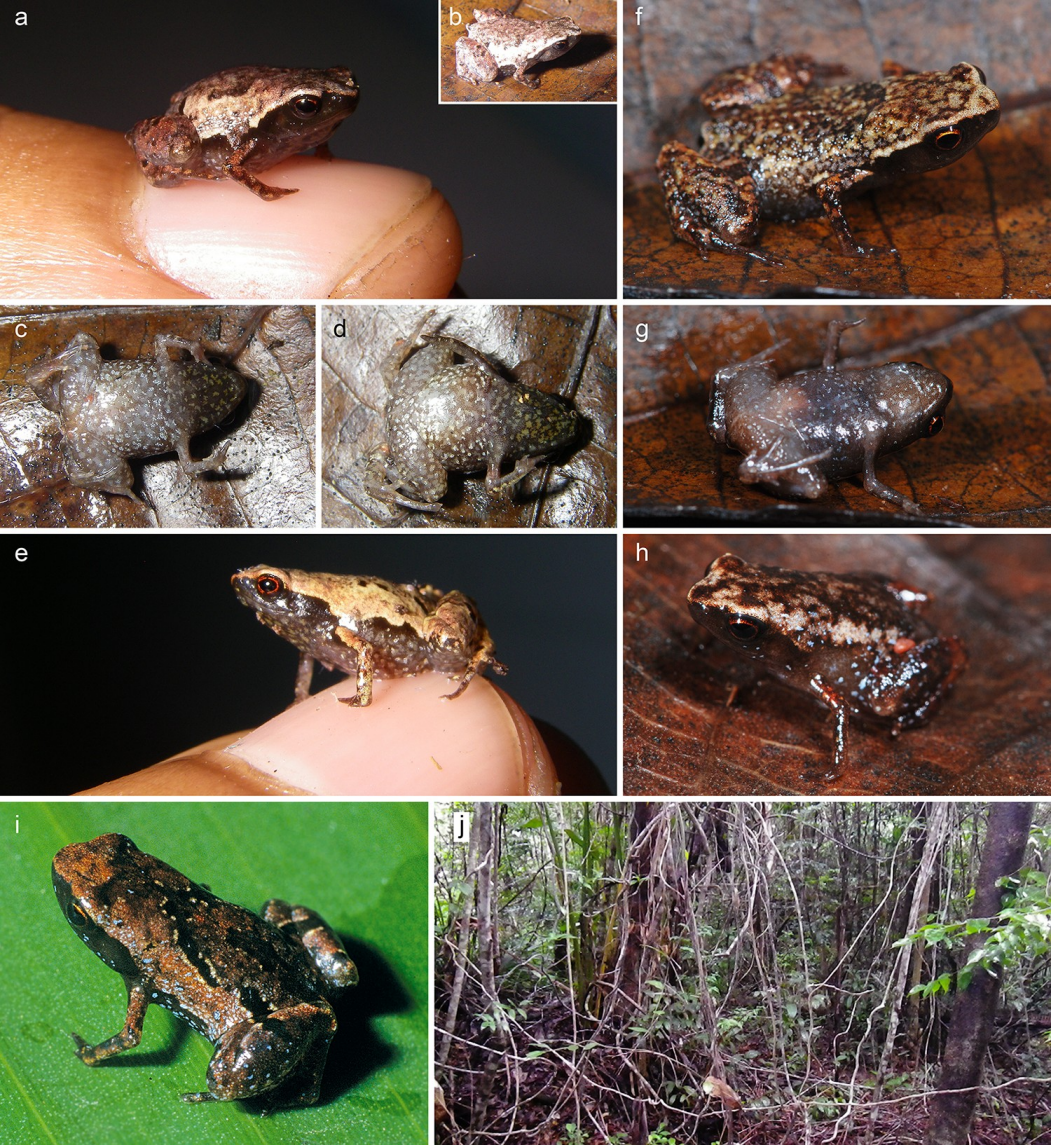
*Mini mum* gen. et sp. nov. in life and its habitat in Manombo Special Reserve. (a-c) ZSM 861/2014, holotype, in (a) anterolateral view on a thumbnail, (b) dorsolateral view on a leaf, (c) ventral view. (d, e) ZSM 862/2014, paratype, in (d) ventral view, and (e) lateral view on a thumbnail. (f, g) ZMB 83194, paratype, in (f) dorsolateral view, and (g) ventral view. (h) ZMB 81993, paratype, in dorsolateral view. (i) ZMA 20172 in posterodorsolateral view. (j) Habitat of the new species in Manombo Special Reserve.

**Fig 5:**
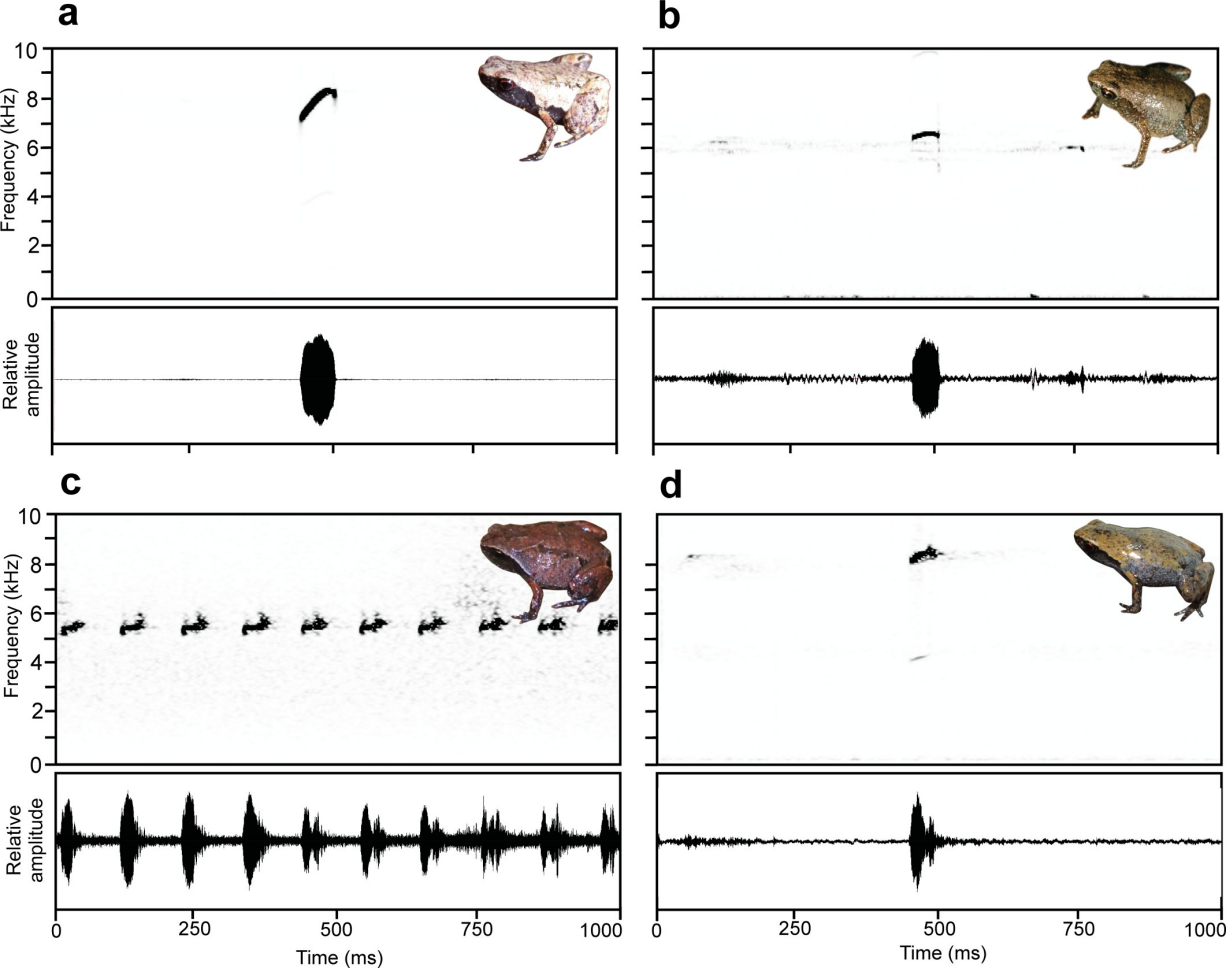
One-second spectrograms and oscillograms of the calls of the new species described here. Insets represent the respective species but not the calling specimens. (a) *Mini mum* gen. et sp. nov., paratype ZMB 81993 from Manombo, (b) *Mini scule* gen. et sp. nov., ZSM 265/2018 from Sainte Luce, (c) *Rhombophryne proportionalis* sp. nov., part of a call (note series) of a specimen from Bepia campsite, Tsaratanana (not collected), (d) *Anodonthyla eximia* sp. nov., specimen not collected, from Maharira (Ranomafana).

**Fig 6:**
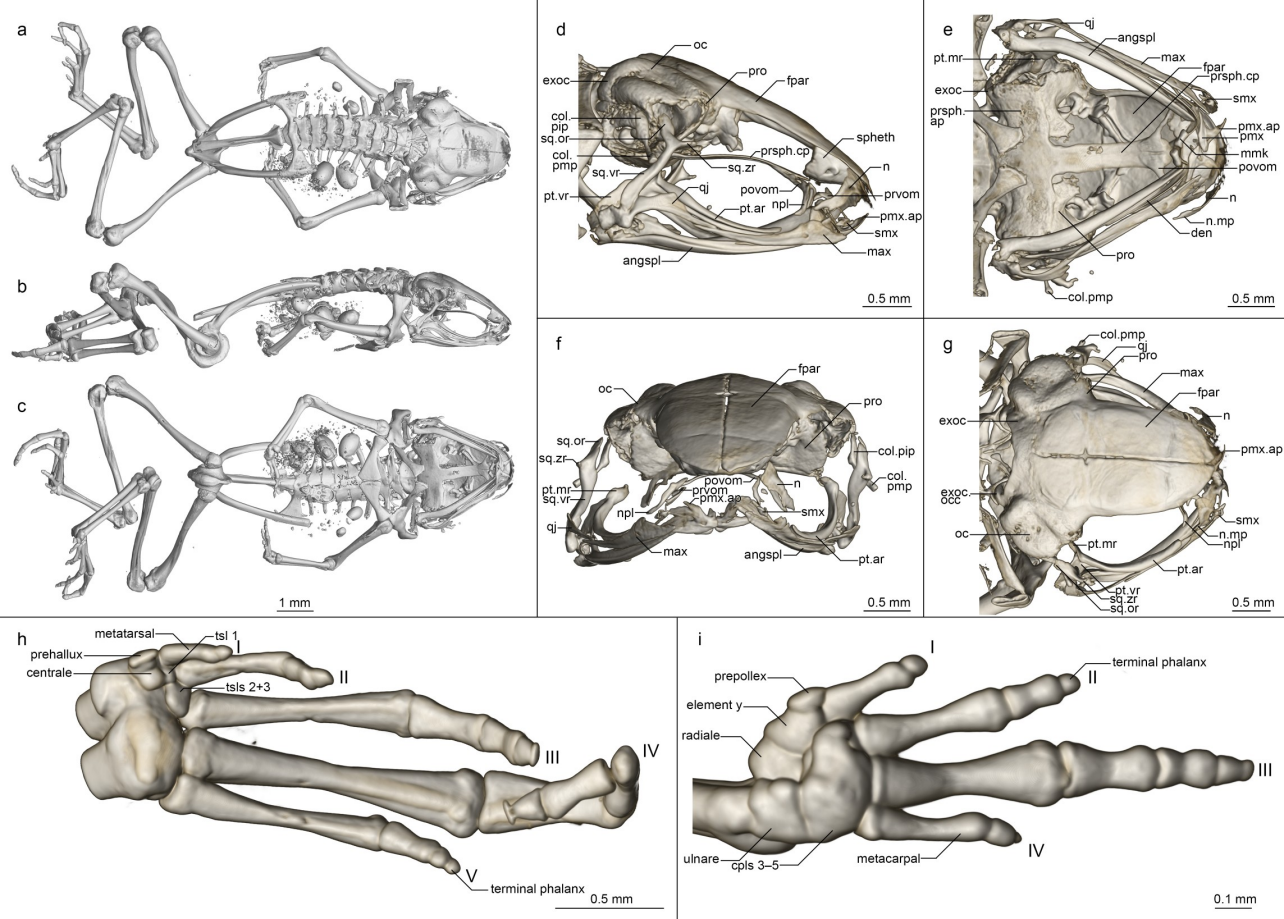
Osteology of *Mini mum* gen. et sp. nov. holotype (ZSM 861/2014). (a-c) Whole skeleton in (a) dorsal, (b) lateral, and (c) ventral view. (d-g) Skull in (d) lateral, (e) ventral, (f) anterior, and (g) dorsal view. (h) Foot in ventral view. (i) Hand in ventral view. Abbreviations for all osteological figures: angspl, angulosplenial; col.pip, pars interna plectra of columella; col.pmp, pars media plectra of columella; cpl(s), carpal(s); den, dentary; exoc, exoccipital; exoc.occ, occipital condyle of exoccipital; fpar, frontoparietal; max, maxilla; max.fp, facial process of maxilla; mmk, mentomeckelian; n, nasal; n.mp, maxillary process of nasal; npl, neopalatine; oc, otic capsule; prsph.ap, parasphenoid alary process; prsph.cp, parasphenoid cultriform process; prsph.pmp, parasphenoid posteromedial process; pmx, premaxilla; pmx.ap, premaxilla ascending process; pmx.lp, premaxilla lateral process; pmx.pp, premaxilla palatine process; povom, postchoanal portion of vomer; pro, prootic; prvom, prechoanal portion of vomer; pt.ar, pterygoid anterior ramus; pt.mr, pterygoid medial ramus; pt.vr, pterygoid ventral ramus; qj, quadratojugal; smx, septomaxilla; spheth, sphenethmoid; sq.or, squamosal otic ramus; sq.vr, squamosal ventral ramus; sq.zr, squamosal zygomatic ramus; tsl(s), tarsals; vt, vomerine teeth.

**Fig 7:**
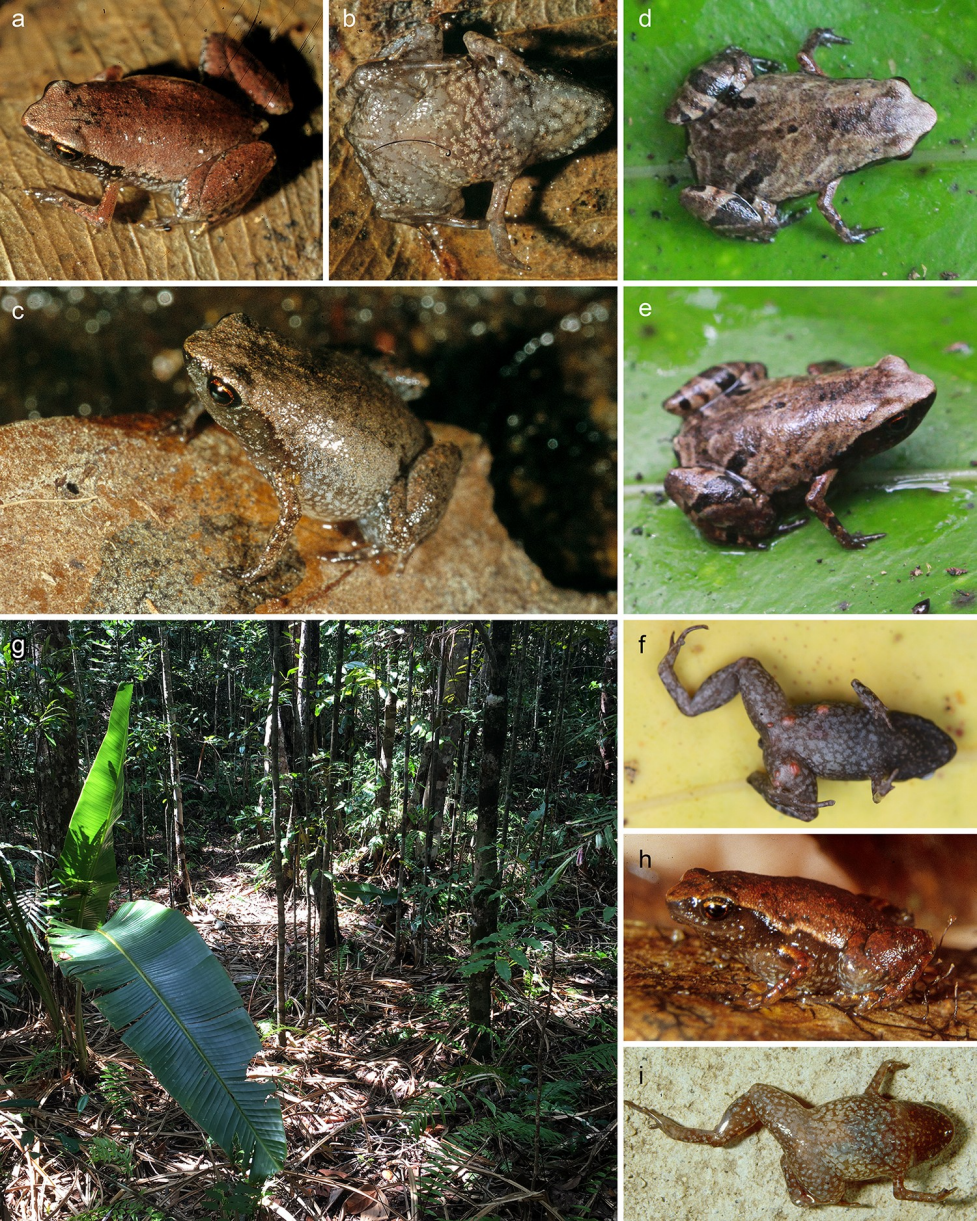
*Mini scule* gen. et sp. nov. in life and its habitat in Sainte Luce Reserve. (a, b) ZSM 5943/2005, holotype, in (a) dorsolateral and (b) ventral view. Black lines in the two pictures are scanning artefacts from damaged analogue slides. (c) Probably ZSM 5942/2005, paratype, in dorsolateral view. (d-f) ZSM 265/2018 (SHR 09112018) in (d) dorsal, (e) dorsolateral, and (f) ventral view. Note the numerous pink cf. *Endotrombicula* mites on the abdomen and legs. (g) Habitat in Sainte Luce Special Reserve. (h, i) *Mini* cf. *scule* from Nahampoana, ZFMK 53775 in (h) lateral and (i) ventral view.

**Fig 8:**
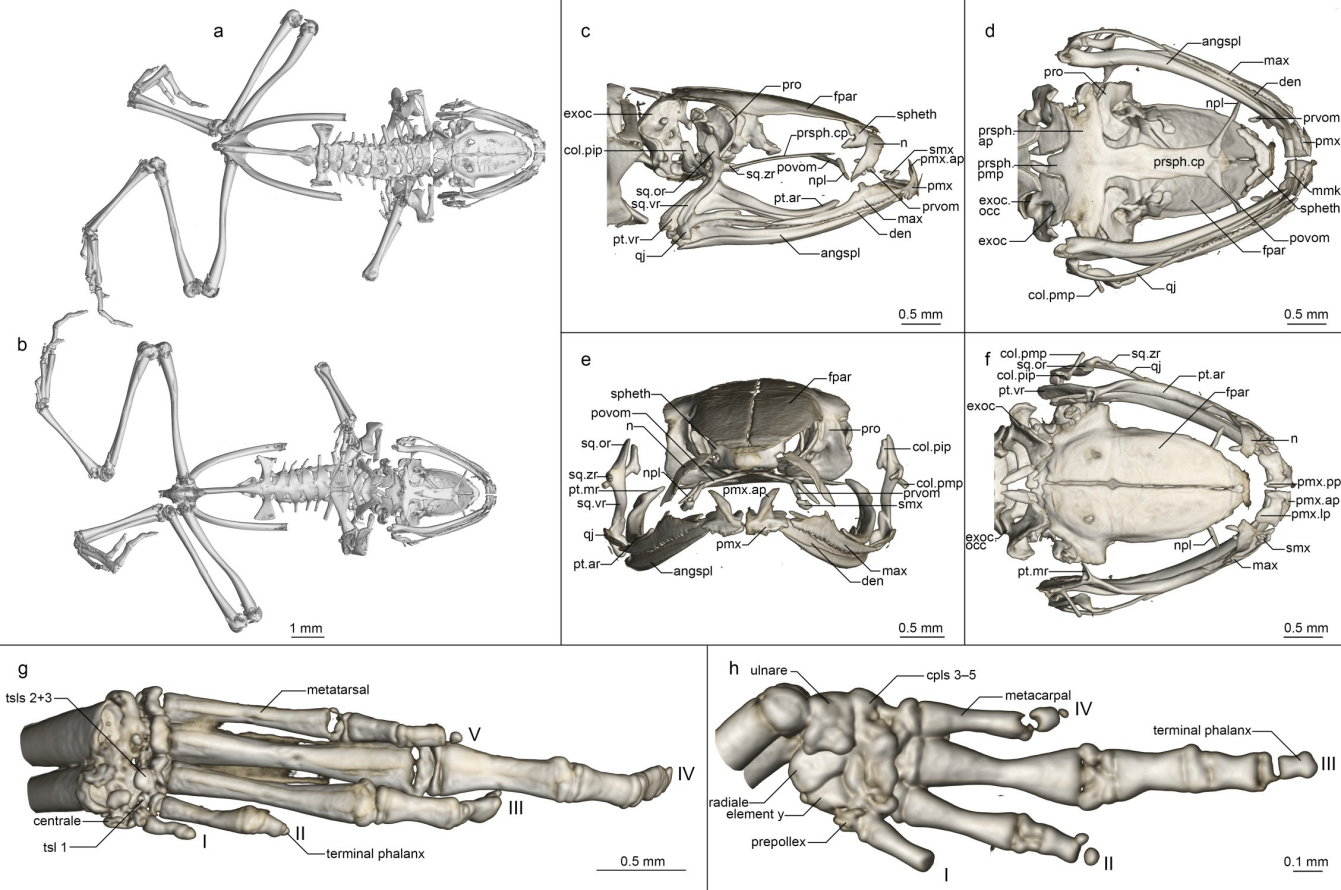
Osteology of *Mini scule* gen. et sp. nov. holotype (ZSM 5943/2005). (a, b) Whole skeleton in (a) dorsal and (b) ventral view. (c-f) Skull in (c) lateral, (d) ventral, (e) anterior, and (f) dorsal view. (g) Foot in ventral view. (h) Hand in ventral view. For abbreviations, see Fig 6.

**Fig 9:**
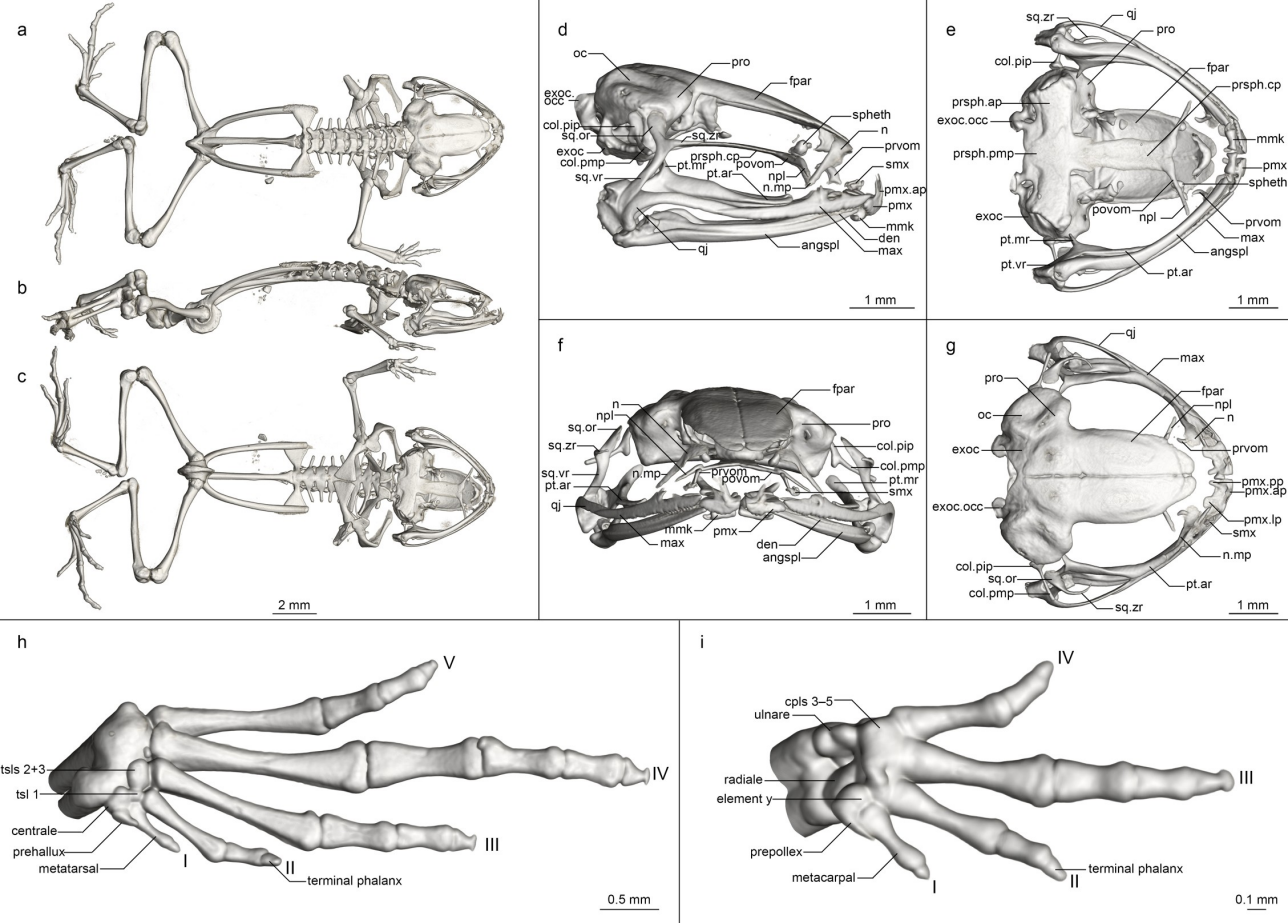
Osteology of *Mini ature* gen. et sp. nov. holotype (ZSM 86/2004). (a-c) Whole skeleton in (a) dorsal, (b) lateral, and (c) ventral view. (d-g) Skull in (d) lateral, (e) ventral, (f) anterior, and (g) dorsal view. (h) Foot in ventral view. (i) Hand in ventral view. For abbreviations, see Fig 6.

**Type species.** *Mini mum* sp. nov.

**Contents.** *Mini mum* sp. nov., *M*. *scule* sp. nov., and *M*. *ature* sp. nov.

**Etymology.** The genus name is derived from English prefix ‘mini-’, denoting a small version of an object. We treat this name as an arbitrary combination of letters in the sense of the International Code of Zoological Nomenclature Articles 30.1.4.1 and 30.2.2, and we assign it the feminine gender. We have searched all available taxonomic databases and could not find any evidence that this name has ever been used to refer to a genus of animals, and we therefore conclude that it is available.

**Diagnosis.** Diminutive terrestrial frogs (adult SVL 8.2–14.9 mm), assigned to the Madagascar-endemic subfamily Cophylinae on the basis of divided vomers, procoelous vertebral column, divided sphenethmoids, and genetic affinities. Skin smooth to slightly granular, occasionally iridescent. A lateral colour border is present but varies in intensity among species. Highly reduced fingers and toes, fusion or loss of carpal 2, and paedomorphic skull morphology: laterally displaced narrow nasals, teeth absent from vomer, in some species present on the maxilla and premaxilla, otic capsule sometimes dorsally ossified, brain case comprising most of the skull’s length and width.

All members of the genus *Mini* gen. nov. resemble miniaturised to extremely miniaturised members of the genus *Stumpffia*. However, all species can be distinguished from *Stumpffia* on the basis of their curving clavicles and a fused or lost carpal 2. In the species accounts below, we provide detailed distinctions from *Stumpffia* relevant to each species.

**Justification.** The erection of the genus *Mini* is justified by significant genetic differentiation from all other major cophyline lineages (see Fig 1), by the fact that it does not form a monophyletic group with the genus *Stumpffia*, and furthermore by the strong morphological differences (including but not restricted to the much smaller size) to all species of its sister clade, *Plethodontohyla*. The following characters distinguish the genus from all *Plethodontohyla* species, including juveniles: digital reduction of the fingers and toes (vs no reduction), laterally displaced and reduced nasals (vs large nasals situated anterior to frontal), parasphenoid cultriform process shorter than frontoparietals (vs roughly equal in length to the frontoparietals) and considerably narrower than alary processes (vs as wide or wider), vomerine teeth absent (vs present), carpal 2 absent (vs present). Uncorrected p-distances between *Mini* and *Plethodontohyla* range from 8.3–13.3% in the 3’ fragment of *16S rRNA* analysed here, and they have been found to be sister to *Plethodontohyla* in all phylogenetic analysis since their first inclusion in genetic datasets [15, 17, 20, 22] (except the DNA barcoding study of Vieites et al. [40], where they were placed at the base of *Rhombophryne+Stumpffia+Anilany*, but that study lacks any resolution at deep nodes, and was not intended to provide phylogenetic hypotheses at deep levels).

**Distribution.** The genus *Mini* is apparently endemic to low-elevation habitats (0–350 m a. s. l.) of southeastern Madagascar (Fig 3).

### *Mini mum* sp. nov.

urn:lsid:zoobank.org:act:237AA825-4612-4591-9CC8-764DAD646B48

(Figs 1–6, Tables 1 and 2)

**Table 2:** Bioacoustic parameters of new species of miniaturised cophyline microhylids. Data on *S*. *miery* from Rakotoarison et al. [14], provided for comparison with the sympatric *A*. *eximia*. Values are presented as mean ± standard deviation, with range in brackets. na = not applicable. *In all species except *R*. *proportionalis* calls consist of a single note according to the definition herein, and in these species call duration is therefore synonymous with note duration.

	Dominant frequency (Hz)	Call duration (ms)*	Inter-call interval (ms)	Note duration (ms)*	Inter-note interval (ms)	Notes per series	Notes analysed*
*Mini mum* gen. et sp. nov.	8089 ± 140 (7676–8306)	74.8 ± 7.0 (57–87)	4299.8 ± 1604.9 (3136–10139)		na	na	n = 35
*Mini scule* gen. et sp. nov. ZSM 265/2018	6675 ± 64 (6549–6768)	121.9 ± 8.7 (108–140)	1905.1 ± 398.3 (1589–4122)		na	na	n = 51
*Rhombophryne proportionalis* sp. nov. (Camp Bepia)	5460 ± 117 (5166–5732)	1328.0 ± 284.1 (905–1765, n = 6)	62753 ± 20613 (38952–74744, n = 3)	45.4 ± 8.2 (27–60)	63.0 ± 9.0 (45–88)	13 ± 3 (9–17, n = 6)	n = 79
*Anodonthyla eximia* sp. nov.	8406 ± 78 (8349–8540)	59.6 ± 6.5 (53–68)	3749.0 ± 1149.9 (2654–5172)		na	na	n = 5
*Stumpffia miery*	8057 ± 137 (7751–8225)	73 ± 12 (51–88)	3102 ± 456 (2679–4247)		na	na	n = 10

**Remark.** This species was previously listed as *Stumpffia* sp. 10 [17]; *Stumpffia* sp. 15/Ca15 [15, 40]; *Stumpffia* sp. aff. *tetradactyla* “Southeast” [41]; and *Stumpffia* sp. 10 KCW-2008 (EU341082) [20].

**Holotype (Figs 2, 4 and 6).** ZSM 861/2014 (ZCMV 14788), an adult presumed male specimen collected in Manombo Special Reserve (23.0294°S, 47.7312°E, 7 m a.s.l.), Atsimo-Atsinanana Region, former Fianarantsoa province, southeastern Madagascar on 30 November 2014 by A. Rakotoarison and E. Rajeriarison.

**Paratypes (Fig 4).** ZSM 862/2014 (ZCMV 14789), an adult presumed male specimen with the same collection data as the holotype; ZMA 20172 (ZCMV 557), an adult presumed male specimen, and ZMA 20191 (ZCMV 558, GenBank accession number EU341082 for *12S rRNA* gene, *tRNA-Val*, and *16S rRNA* gene), an unsexed specimen collected in Manombo Special Reserve (23.0284°S, 47.7316°E, 44 m a.s.l.) on 2 February 2004 by D.R. Vieites and C. Woodhead; ZMB 81993 (NSH 2584) and ZMB 83194 (NSH 2583), adult male and female specimens (respectively) collected in Manombo Special Reserve (23.0249°S, 47.7311°E, ca. 20 m a.s.l.) on 28 March 2012 by J.C. Riemann, S.H. Ndriantsoa, A. Rakotoarison, J. Glos and M.-O. Rödel.

**Diagnosis.** An extremely miniaturised frog assigned to *Mini* gen. nov. on the basis of its small size, curved clavicles, laterally displaced and reduced nasals, and fusion or loss of carpal 2. This assignment is supported by its genetic affinities (Fig 1; [15, 17, 20]). It is separated by uncorrected p-distances of 10.0–11.2% in the analysed 3’ fragment of the *16S rRNA* gene from other members of the genus *Mini* gen. nov., and 8.3–12.4% from all members of the genus *Plethodontohyla*.

*Mini mum* sp. nov. is characterised by the unique combination of the following characters (n = 4 male specimens, 1 female specimen): (1) male SVL 8.2–9.7 mm, female SVL 11.3 mm; (2) ED/HL 0.38–0.56; (3) HW/SVL 0.28–0.37; (4) FARL/SVL 0.30–0.38; (5) TIBL/SVL 0.39–0.50; (6) HIL/SVL 1.47–1.72; (7) fingers 1, 2, and 4 strongly reduced; (8) toe 1 absent, toes 2 and 5 quite reduced; (9) maxillary and premaxillary teeth absent; (10) vomerine teeth absent; (11) strong lateral colour border present; (12) black inguinal spots absent; (13) postchoanal vomer present, spatulate, medially fused to parasphenoid; (14) nasal cultriform and laterally displaced; (15) quadratojugal-maxilla contact weak; (16) zygomatic ramus of squamosal short, narrow, and horizontal; (17) clavicles present, curving with simple lateral articulations, medially not bulbous; (18) prepollex small or absent; (19) carpal 2 absent or fused to post-axial carpal 3–5 element; (20) finger phalangeal formula 1-2-3-2; (21) toe phalangeal formula 1-2-3-4-3; (22) single-note, unpulsed calls, not emitted in series; (23) frequency modulated calls; (24) call dominant frequency 8089 ± 140 Hz (n = 35); (25) call duration 74.8 ± 7.0 ms (n = 35); (26) inter-call interval 4299.8 ± 1604.9 ms (n = 34).

Within the genus *Mini* gen. nov., the new species is unique in lacking teeth, and possessing a strong lateral colour border. See other species described below for respective diagnoses.

This species is particularly similar to some extremely miniaturised *Stumpffia* species, but it can be distinguished from all *Stumpffia* based on the condition of the carpals, and all *Stumpffia* except *S*. *tridactyla*, *S*. *contumelia*, and *S*. *obscoena* by the extent of reduction of its fingers and toes. It differs from all of these in possessing curved clavicles (vs absent in *S*. *contumelia* and *S*. *obscoena* and straight or absent in *S*. *tridactyla*; unpublished data), and presence of neopalatine and divided vomer (vs absence of neopalatine and non-divided vomer in *S*. *obscoena*, *S*. *tridactyla*, and *S*. *contumelia*; unpublished data).

Calls resemble those of *S*. *miery*, *S*. *tridactyla*, *S*. *contumelia*, and *S*. *obscoena*, but are shorter in duration and lower in frequency than *S*. *obscoena* (duration 74.8 ± 7.0 ms vs 144 ± 8 ms; frequency 8089 ± 140 Hz vs 8361 ± 69 Hz); longer in duration and higher in frequency than *S*. *contumelia* (duration 74.8 ± 7.0 ms vs 42 ± 4 ms; frequency 8089 ± 140 Hz vs 7493 ± 50 Hz); shorter in duration and higher in frequency than *S*. *tridactyla* (duration 74.8 ± 7.0 ms vs 132 ± 23 ms; frequency 8089 ± 140 Hz vs 7244 ± 200 Hz); and slightly longer inter-call interval than *S*. *miery* (4299.8 ± 1604.9 ms vs 3102 ± 456 ms).

**Holotype description.** Specimen in a good state of preservation, a piece of the left thigh removed as a tissue sample. Body oblong; head wider than long, narrower than body width; snout rounded in dorsal view, pointed in lateral view; nostrils directed laterally, not protuberant, slightly further from tip of snout than from eye; canthus rostralis indistinct, straight; loreal region flat, vertical; tympanum indistinct, round, about 49% of eye diameter; round pupil; supratympanic fold absent; tongue long, broadening slightly posteriorly, attached anteriorly, not notched; maxillary teeth absent; vomerine teeth absent; choanae small and round. Forelimbs slender; subarticular tubercles single, indistinct except on third finger; outer metacarpal tubercle rounded; inner metacarpal tubercle indistinguishable from reduced first finger; hand without webbing; first, second, and fourth fingers strongly reduced, third finger basally broadened; relative length of fingers 1<4<2<3, fourth finger slightly more reduced than second; finger tips not expanded into discs. Hindlimbs slender; TIBL 50% of SVL; lateral metatarsalia strongly connected; inner metatarsal tubercle indistinguishable from completely reduced first toe; outer metatarsal tubercle absent; no webbing between toes; first toe absent, second and fifth toes extremely reduced; relative length of toes 2<5<3<4; fifth toe distinctly shorter than third. Skin on dorsum smooth, without distinct dorsolateral folds. Ventral skin smooth.

After four years in 70% ethanol, the dorsum is metallic silver centrally on the trunk, bluish silver on the head, and laterally light silver, with dark oblong markings in the inguinal region (Fig 2). There is a strong dorsolateral colour border to the ebony lateral colouration, extending from the side of the head to the legs. The lateral colouration fades to the more burnt umber ventral colouration, especially dark anteriorly, flecked with beige, fading posteriorly through larger fleck sizes to beige at the posterior abdomen. Dorsally, the legs are mottled cream and grey brown with a dark cloacal region. Ventrally the legs are brown flecked with beige. The arms are silvery dorsally and ebony laterally and ventrally. Colour in life (Fig 4A–4C) as in preservative but browner in every aspect and less obviously iridescent, with a red iris.

**Variation.** For variation in measurements among specimens, see Table 1. Non-ovigerous specimens with darkened throats are presumed to be males, in keeping with the one call voucher, ZMB 81993. In general, all examined specimens agree with the holotype in morphology, but female ZMB 83194 is more rotund in body shape in preservative, but had a longer, depressed body profile in life (Fig 4F and 4G). ZMB 83194 and 83193 varied in life from smooth to slightly granular skin, with a very faintly bulging vertebral line. The colouration in life varied rather strongly (Fig 4). Lateral and ventral colouration was more or less consistently dark brown with light flecks, but ZMB 81993 had bright bluish-white flecks laterally. The strength of the flank colour border varied from stark in ZSM 862/2014 to weak in ZMB 83194. Dorsal colouration varied from solid tan in ZSM 862/2014 to mottled beige and dark brown in ZMB 83194. Iris colouration was consistently red to reddish copper. In preservative, paratypes are less iridescent than the holotype, and ZMB 81993 and 83914 are faded to light brown.

**Bioacoustics.** Calls recorded from specimen ZMB 81993 (Fig 5A, Table 2) during the day on 28 March 2012 (see paratype section for locality data). Air temperature was 21.7°C. The specimen was calling under dense leaf litter. Estimated call parameters were as follows (n = 35 in all cases except inter-call and call intervals, where n = 34): Calls consisting of a single note were emitted at regular intervals without defined call series. Calls had linear upward frequency modulation with a downward hooked tail, with an initial dominant frequency around 7000 Hz, rising gradually to ca. 8250 Hz, and with a tail-end dropping down to ca. 7500 Hz again. For detailed parameters, see Table 2.

**Osteology (Fig 6).** Based on ZSM 861/2014 (figured), and ZSM 862/2014, ZMA 20172, ZMB 81993 and ZMB 83194 (not figured). Note that the skulls of ZSM 861–862/2014 are somewhat distorted in fixation, especially with respect to the maxillary arcade and mandible. The skull and pectoral girdle of ZMB 81993 are quite badly damaged, and both of its hindlimbs are fractured, as are the coracoids and left ilium of ZMB 83194.

**Cranium (Fig 6D–6G).** Shape and proportions. Skull short and rounded, longer than wide, widest at the bowing of the quadratojugal roughly in line with the anterior face of the prootic. Braincase proportionally broad, with an extremely short rostrum.

**Neurocranium.** Ossification generally high, lower in ZSM 862/2014 than other specimens. Anterior cone of sphenethmoid ossified and in contact with the frontoparietals in ZSM 861/2014 and ZMB 83194 and 81993, but no sphenethmoid ossification in the other specimens. Prootic in dorsal contact with lateral flange of frontoparietal, ventral contact with parasphenoid alae, not approaching contralateral ventrally. Septomaxilla miniscule, very tightly curled, not further discussed due to low ossification and insufficient resolution. Columella (stapes) well ossified, pars media plectra (stylus) long and nearly straight, broadening distally, posteriorly and dorsally oriented toward the dorsally elongated pars interna plectra (baseplate). Nasal narrow and cultriform, laterally displaced (in line with anterior end of frontoparietal), curved downward laterally, the acuminate maxillary process not distinct and not closely approaching maxillary pars facialis, broadly separated from contralateral. Frontoparietal with rounded anterior edge, laterally rather straight-edged, with short lateral flange covering prootic, posteriorly strongly connected to exoccipital, anteroventrally contacting sphenethmoid in ZSM 861/2014, lacking any dorsal process, separated from contralateral by a narrow gap with a small rhomboid facet at the level of the prootics, possibly constituting the pineal foramen.

Parasphenoid with narrow, rather straight-edged cultriform process and slightly broader perpendicular alae, considerably shorter than frontoparietals, in contact with exoccipitals posterodorsally, prootics dorsally along the edges of the alae, anteroventrally in contact with postchoanal vomer and not in contact with neopalatine; posteromedial process not participating in foramen magnum. Vomer divided into pre- and postchoanal portions; prechoanal portion narrow, simple, sickle-shaped, without a lateral ramus; postchoanal portion spatulate and edentate, narrowly separated from its contralateral on the midline, in dorsal contact with the parasphenoid proximally and the neopalatine distally, lacking an anterior projection. Neopalatine simple, straight, almost indistinguishable from lateral postchoanal portion of vomer, laterally broadly separated from the maxilla, not exceeding the lateral-most point of the postchoanal vomer.

Maxillary arcade gracile, maxilla and premaxilla edentate, anterior extension of maxilla not exceeding lateral extent of premaxilla. Premaxilla with a narrow acuminate dorsal alary process rising laterally, pars palatina shallowly divided into a narrow palatine process and broad, squared lateral process. Maxilla with a low triangular pars facialis and a narrow pars palatina, its posterior tip acuminate and barely contacting the quadratojugal, the lingual surface of the pars palatina followed by but not contacting the anterior ramus of the pterygoid, presumably separated by the pterygoid cartilage. Pterygoid with an exceptionally short medial ramus, long anterior ramus, and broad posterior ramus, posteriorly weakly calcified to the quadratojugal complex. Quadratojugal bowed laterally, broadly connected to the ventral ramus of the squamosal, bearing a small posteroventral knob, weakly anteriorly connected to the maxilla; the articulation of the mandible is apparently somewhat fortified by the mineralisation of the posterior ramus of the pterygoid+squamosal+quadratojugal posteroventral knob. Squamosal with a slender, rather straight ventral ramus, broadened, nearly vertical otic ramus, and short, thin, horizontal zygomatic ramus.

Mandible slim and edentate, largely unremarkable, with a weakly raised coronoid process on the angulosplenial. Mentomeckelians separated from the dentary, with small hooked ventrolateral projections.

Posteromedial processes of hyoid proximally rounded with a broad medial crista.

**Postcranial Skeleton (Fig 6A–6C, 6H and 6I).** Eight procoelous presacrals, all much broader than long, lacking neural spines, with round posterior articular processes, presacral I with a complete neural arch, presacrals II–IV with thicker and longer transverse processes than V–VIII. Sacrum with expanded diapophyses, the leading and trailing edges roughly equally angled, the articulation type IIB *sensu* Emerson [42]. Urostyle bicondylar, long, broadening posteriorly, with a somewhat flared head and a low dorsal ridge.

Pectoral girdle without ossified prezonal or postzonal elements, with ossified clavicles. Clavicle thin and weakly curved, with a simple lateral junction, slightly shorter than the coracoid. Coracoid fairly narrow, weakly flared laterally, strongly flared medially with a straight medial articular surface with the contralateral. Scapula slender, with a thin pars acromialis, the cleitheral border straight. Cleithrum ossified for half the width of the scapular border, thickened anteriorly. Suprascapula unossified.

Humerus with a well-developed crista ventralis and no medial or lateral cristae. Radioulna slender with a distinct sulcus intermedius. Carpals apparently reduced, composed of radiale, ulnare, element Y, prepollex, and a large post-axial element formed by carpals 3–5. Carpal 2 has either been lost or fused to the latter element. Finger phalangeal formula is reduced (1-2-3-2), and the terminal phalanges of the first, second and fourth fingers are small, round elements.

Pubis ossified; iliac shafts passing ventral to and beyond sacrum, oblong in cross-section, with a weak dorsal crest, without a dorsal prominence and with a shallow oblique groove. Femur weakly sigmoid, lacking a posterior crest. Tibiofibula slightly longer than femur in length, with a sulcus intermedius. Tibiale and fibulare fused proximally and distally. T1 and T2+3 tarsals present, T1 considerably smaller than T2+3. Centrale present, slightly smaller than T2+3. Prehallux small. Phalangeal formula reduced (1-2-3-4-3). Terminal phalanges of toes 3 and 4 with knobs, those of other toes small, round elements.

**Distribution, natural history, and conservation status.** This species is known only from Manombo Special Reserve, southeast Madagascar (Fig 3). The habitat consists of low, comparatively open forest with small trees, many lianas and a very thick layer of dead leaves (Fig 4J). Calls were emitted by males during the day, hiding within the leaf litter or between roots, separated from other calling males by several metres. The female paratype ZMB 83194 contains four eggs (visualised by micro-CT scan, not extracted for physical examination). Manombo Special Reserve covers an area of 52.66 km^2^. We estimate that this species occurs from 0–100 m a.s.l. within lowland forests in and around this reserve. Although lowland species from areas with low topographical complexity tend not to be extreme micro-endemics [43], extremely miniaturized frogs in Madagascar almost always are [14, 17], so the full extent of this species’ range is not likely to be large. Littoral forest in the area where the species occurs is extremely reduced, so other sites outside the Special Reserve are likely to be small and under high pressure. We therefore recommend this species be listed as Critically Endangered according to the IUCN Red List Criterion CR B1ab(iii) [44], in line with other endemics from Manombo Special Reserve (e.g. *Guibemantis diphonus* [45]).

The gut of ZSM 861/2014 contains four or five arthropods visible from micro-CT scans, tentatively identified as oribatid mites.

**Etymology.** We use the specific epithet ‘mum’ as an arbitrary combination of letters, in order to form a pun on ‘minimum’ from the name in apposition, in reference to the fact that this is among the smallest known frogs from Madagascar and the world. It is to be regarded as an invariable noun.

### *Mini scule* sp. nov.

urn:lsid:zoobank.org:act:AC570728-78AE-4FDB-B9BE-87DB553C5ABE

(Figs 1–3, 7 and 8, Tables 1 and 2)

**Remarks.** This species was previously listed as *Stumpffia* sp. 9 [17]; *Stumpffia* sp. 16/Ca16 [15, 40, 46]; *Stumpffia* sp. aff. *tetradactyla* “Southeast” [41]; and *Stumpffia* sp. 16 MV-2009 (KC351485) [20].

ZFMK 53775 (Fig 7D and 7E), a specimen from Nahampoana in southeastern Madagascar (ca. 24.975°S, 46.980°E, ca. 60 m a.s.l.), collected by F. Glaw and J. Müller on 4 January 1992, is similar to *M*. *scule* sp. nov. and we consider it possible that it is a member of this species. However, in the absence of genetic data from this specimen and population, we here do not consider it within the definition of *M*. *scule* sp. nov. and refer to it as *M*. cf. *scule*.

**Holotype (Figs 2, 7 and 8).** ZSM 5943/2005 (FGZC 2662, GenBank accession number KC351485 for *16S rRNA* gene), an adult presumed male specimen collected in Sainte Luce Reserve forest at the QMM climate station (24.7798°S, 47.1713°E, 23 m a.s.l.), Anosy Region, former Toliara province, southeastern Madagascar on 4 February 2005 by F. Glaw and P. Bora.

**Paratype (Fig 7).** ZSM 5942/2005 (FGZC 2661, GenBank accession number EU341081 for *12S rRNA* gene, *tRNA-Val*, and *16S rRNA*e genes), an adult presumed male specimen with the same collection data as the holotype. The hands and feet of this specimen were all removed as tissue samples. ZSM 265/2018 (SHR 09112018), an adult male specimen collected while calling in Sainte Luce Reserve parcel S9 (24.7606°S, 47.1732°E, 28 m a.s.l.) on 8 November 2018 by S. Hyde Roberts. Additionally, the following five specimens collected by S. Hyde Roberts between 8 and 20 October 2016 in Sainte Luce Reserve: UADBA-A Uncatalogued (ACZCV 0386, GenBank accession number MK459315 for *16S rRNA* gene) an unsexed adult specimen, and UADBA-A Uncatalogued (ACZCV 0387, GenBank accession number MK459316 for *16S rRNA* gene), a juvenile female specimen (sexed by incision, small dark brown egg follicles present), both collected at 24.754–24.755°S, 47.173°E, ca. 20 m a.s.l.; ZSM 577/2016 (ACZCV 0383, GenBank accession number MK459312 for *16S rRNA* gene), an adult unsexed specimen collected at 24.7600°S, 47.1746°E, ca. 20 m a.s.l.; UADBA-A Uncatalogued (ACZCV 0384, GenBank accession number MK459313 for *16S rRNA* gene), an adult male specimen collected at 24.7604°S, 47.1737°E, ca. 20 m a.s.l.; ZSM 578/2016 (ACZCV 0385, GenBank accession number MK459314 for *16S rRNA* gene), an adult unsexed specimen collected at 24.7550°S, 47.1735°E, ca. 20 m a.s.l.

**Diagnosis.** An extremely miniaturised frog assigned to *Mini* gen. nov. on the basis of its small size, curved clavicles, laterally displaced and reduced nasals, and fusion or loss of carpal 2. This assignment is supported by its genetic affinities (Fig 1; [15, 17, 20]). It is separated by uncorrected p-distances of 10.4–11.2% in the analysed 3’ fragment of the *16S rRNA* gene from other members of the genus *Mini* gen. nov., and 9.7–13.3% from all members of the genus *Plethodontohyla*.

*Mini scule* sp. nov. is characterised by the unique combination of the following characters (n = 3 probable male and 3 adult unsexed specimens): (1) male SVL 9.9–10.5 mm (adult SVL up to 10.8 mm); (2) ED/HL 0.40–0.51; (3) HW/SVL 0.31–0.38; (4) FARL/SVL 0.34–0.39; (5) TIBL/SVL 0.39–0.47; (6) HIL/SVL 1.41–1.68; (7) fingers 1, 2, and 4 strongly reduced; (8) toe 1 absent, toes 2 and 5 quite reduced; (9) maxillary and premaxillary teeth present; (10) vomerine teeth absent; (11) lateral colour border occasionally present; (12) black inguinal spots generally absent; (13) postchoanal vomer present, spatulate, medially fused to parasphenoid; (14) nasal cultriform and laterally displaced; (15) quadratojugal-maxilla contact weak; (16) zygomatic ramus of squamosal short, thick, and horizontal; (17) clavicles present, curving with simple lateral articulations, medially not bulbous; (18) prepollex small or absent; (19) carpal 2 absent or fused to post-axial carpal 3–5 element; (20) finger phalangeal formula 0-2-3-2; (21) toe phalangeal formula 1-2-3-4-3; (22) single-note, unpulsed calls, not emitted in series; (23) non-frequency modulated calls; (24) call dominant frequency 6675 ± 64 Hz (n = 51); (25) call duration 121.9 ± 8.7 ms (n = 51); (26) inter-call interval 1905.1 ± 398.3 ms (n = 50).

Within the genus *Mini* gen. nov., the new species can be distinguished from *M*. *mum* sp. nov. by the presence of maxillary and premaxillary teeth (vs absence), and less distinct lateral colour border. For diagnosis against *M*. *ature* sp. nov., see the diagnosis of that species, below.

This species is particularly similar to some extremely miniaturised *Stumpffia* species, but it can be distinguished from all *Stumpffia* based on the condition of the carpals, from almost all *Stumpffia* by the possession of maxillary and premaxillary teeth (present only in *S*. *spandei*, *S*. *miovaova*, *S*. *makira*, *S*. *diutissima*; unpublished data), and from all *Stumpffia* except *S*. *tridactyla*, *S*. *contumelia*, and *S*. *obscoena* by the extremely reduced fingers and toes. It differs from these latter three species in lacking a strong lateral colour border (vs present), curved clavicles (vs absent in *S*. *contumelia* and *S*. *obscoena* and straight or absent in *S*. *tridactyla*; unpublished data), and presence of neopalatine and divided vomer (vs absence of neopalatine and non-divided vomer in *S*. *obscoena*, *S*. *tridactyla*, and *S*. *contumelia*; unpublished data).

Calls differ significantly from *M*. *mum* sp. nov. in frequency, duration, and inter-call intervals (see Table 2), but resemble those of numerous *Stumpffia* species. For distinction, compare the values given in Table 5 of Rakotoarison et al. [14]. In call duration, the calls are most similar to *S*. *gimmeli*, *S*. *larinki*, and *S*. *tridactyla*, but they are higher in dominant frequency than *S*. *gimmeli* and *S*. *larinki* (6675 ± 64 Hz vs 4823 ± 302 Hz in *S*. *gimmeli* and 2914 ± 124 Hz in *S*. *larinki*), and lower in dominant frequency with a longer inter-call interval than *S*. *tridactyla* (dominant frequency 6675 ± 64 Hz Hz vs 7244 ± 200 Hz; inter-call interval 1905.1 ± 398.3 ms vs 1012 ± 39 ms).

**Holotype description.** Specimen in a moderately good state of preservation, the left arm removed as a tissue sample. Body oblong; head wider than long, narrower than body width; snout rounded in dorsal view, squared in lateral view; nostrils directed laterally, not protuberant, equidistant between tip of snout and eye; canthus rostralis indistinct, straight; loreal region flat, vertical; tympanum indistinct, round, about 55% of eye diameter; supratympanic fold absent; tongue long and thin, attached anteriorly, not notched; maxillary teeth present; vomerine teeth absent; choanae small and round, located very far forward. Forelimbs slender; subarticular tubercles single, indistinct; outer/palmar metacarpal tubercle rounded; inner metacarpal tubercle small and indistinct; hand without webbing; first, second, and fourth fingers strongly reduced, third finger basally broadened; relative length of fingers 1<4<2<3, fourth finger slightly more reduced than second; finger tips not expanded into discs. Hind limbs slender; TIBL 43% of SVL; lateral metatarsalia strongly connected; inner metatarsal tubercle indistinguishable from completely reduced first toe; outer metatarsal tubercle absent; no webbing between toes; first toe absent, second and fifth toes extremely reduced; relative length of toes 2<5<3<4; fifth toe distinctly shorter than third. Skin on dorsum smooth, without distinct dorsolateral folds. Ventral skin smooth.

After 12 years in 70% ethanol, the dorsum is metallic silver over the whole body, excepting brown colour in the inguinal region and the posterior surface of the thigh (Fig 2). There is a moderately distinct colour border between the dorsal and ventral colouration that runs the length of the flank. The side of the head is dark brown, but this becomes increasingly flecked with cream posteriorly. The ventral and lower lateral colouration is cream flecked with brown, most densely on the anterior abdomen, and most loosely at the posterior abdomen. This flecking becomes akin to ocelli on the ventral surfaces of the legs. Colour pattern in life was the same as in preservative, but dorsal colouration was bronze instead of silver (compare Fig 7A and 7B with Fig 2). Iris was rust red.

**Variation.** For measurements, see Table 1. The paratypes strongly resemble the holotype in morphology. Colouration among paratypes is highly variable. ZSM 5942/2005 resembles the holotype but is steelier in colour (Fig 7C). Dark markings in the inguinal region are present in ZSM 5942/2005 and ZSM 265/2018, and both specimens have a more distinctly black flank than the holotype (Fig 7C–7F). ZSM 265/2018 additionally has broad burnt umber crossband on its thighs and shanks.

**Bioacoustics.** Calls recorded from ZSM 265/2018 by S. Hyde Roberts (Fig 4B, Table 2) on 8 November 2018 at 10h16, at an air temperature of 30.4°C. The individual was found in forest habitat (parcel S9 at 24.7606°S, 47.1732°E, 28 m a.s.l.), ca. 3 m from a lentic stream, with an estimated canopy height of 11 m and canopy cover of ~70% after a night of heavy rain. Call details (n = 51 in all cases except inter-call intervals, where n = 30): Calls consisted of a single note and were emitted at regular intervals without defined call series. Calls were not or only very slightly frequency modulated. For detailed call parameters, see Table 2.

**Osteology (Fig 8).** Based on ZSM 5942/2005 (not figured) and ZSM 5943/2005 (figured).

**Cranium (Fig 8C–8F).** Shape and proportions. Skull narrow, longer than wide, widest at the level of the dorsal end of the squamosal and the anterior edge of the otic capsule. Braincase proportionally broad, with a short rostrum.

**Neurocranium.** Ossification varies: highly ossified in ZSM 5942/2005 with ossified otic capsules, less ossified in ZSM 5943/2005, without otic capsule ossification. Only the anterior cone of the sphenethmoid is ossified and contacts the frontoparietal dorsally but is not in contact with any other bones. Prootic in dorsal contact with lateral flange of frontoparietal, ventral contact with parasphenoid alae, not approaching contralateral ventrally. Septomaxilla miniscule, very tightly curled, with a long and thin posterior ramus. Columella (stapes) well ossified, pars media plectra (stylus) long and nearly straight, posteriorly and dorsally oriented toward the elongated pars interna plectra (baseplate). Nasal narrow and cultriform, laterally displaced, curved downward laterally, the acuminate maxillary process not closely approaching maxillary pars facialis, broadly separated from contralateral. Frontoparietal with rounded anterior edge, laterally rather straight-edged, with short lateral flange covering prootic, posteriorly strongly (ZSM 5942/2005) or weakly (ZSM 5943/2005) connected to exoccipital, anteroventrally contacting sphenethmoid, lacking any dorsal process, separated from contralateral by a narrow gap, with a clear, rhomboid facet at the level of the prootics, which may represent a pineal foramen.

Parasphenoid with narrow, rather straight-edged cultriform process and slightly broader posterior-curved alae, considerably shorter than frontoparietals, in contact with exoccipitals posterodorsally, prootics dorsally along the edges of the alae, anteroventrally in contact with postchoanal vomer and not in contact with neopalatine; posteromedial process not participating in foramen magnum. Vomer divided into pre- and postchoanal portions; prechoanal portion narrow, simple, curved, with a suggestion of a lateral ramus; postchoanal portion spatulate and edentate, narrowly separated from its contralateral on the midline, in dorsal contact with the parasphenoid proximally and the neopalatine distally, lacking an anterior projection. Neopalatine simple, straight, weakly distinguishable from lateral postchoanal portion of vomer, laterally broadly separated from the maxilla, not exceeding the lateral-most point of the postchoanal vomer.

Maxillary arcade gracile, maxilla and premaxilla bearing numerous small teeth, anterior extension of maxilla exceeding lateral extent of premaxilla but not in contact with it. Premaxilla with a narrow acuminate dorsal alary process rising laterally, pars palatina shallowly divided into a narrow palatine process and broad lateral process. Maxilla with a low triangular pars facialis and a narrow pars palatina, its posterior tip acuminate and barely contacting the quadratojugal, the lingual surface of the pars palatina in contact with the anterior ramus of the pterygoid. Pterygoid with an exceptionally short medial ramus, long anterior ramus, and broad posterior ramus, posteriorly calcified to the quadratojugal complex. Quadratojugal weakly bowed laterally, broadly connected to the ventral ramus of the squamosal, bearing a small posteroventral knob, weakly anteriorly connected to the maxilla; the articulation of the mandible is apparently fortified by the mineralisation of the posterior ramus of the pterygoid+squamosal+quadratojugal posteroventral knob. Squamosal with a slender, sigmoid ventral ramus, broadened otic ramus, and short, thick zygomatic ramus, the otic ramus oriented dorsally and posteriorly, the zygomatic ramus horizontal.

Mandible slim and edentate, largely unremarkable, with a moderately raised coronoid process on the angulosplenial. Mentomeckelians separated from the dentary, with slightly bulbous, almost hooked ventrolateral projections sometimes present (present in ZSM 5942/2005, absent in ZSM 5943/2005).

Posteromedial processes of hyoid proximally rounded with a broad medial crista.

**Postcranial skeleton (Fig 8A, 8B, 8G and 8H).** Eight procoelous presacrals, with some differentiation errors in ZSM 5942/2005 leading to a transverse process forming on the head of the urostyle; all presacrals much broader than long, lacking neural spines, with round posterior articular processes, presacral I with a more or less complete neural arch, presacrals II–IV with thicker and longer transverse processes than V–VIII. Sacrum with expanded diapophyses, the leading and trailing edges roughly equally angled, the articulation type IIB *sensu* Emerson [42]. Urostyle bicondylar, long, not broadening posteriorly, with a somewhat flared head in ZSM 5943/2005 and with a low dorsal ridge.

Pectoral girdle without ossified prezonal or postzonal elements, with ossified clavicles, badly fractured in ZSM 5942/2005. Clavicle thin and weakly curved, with a simple lateral junction, shorter than the coracoid. Coracoid fairly narrow, not flared laterally, strongly flared medially with a curved medial articular surface with the contralateral. Scapula slender, with a thin pars acromialis, the cleithral border straight. Cleithrum ossified for two thirds the width of the scapular border, thickened anteriorly. Suprascapula unossified.

Arms and legs described only from ZSM 5943/2005, as the limbs of ZSM 5942/2005 were removed for DNA sequencing. Humerus with a well-developed crista ventralis and no medial or lateral cristae. Radioulna slender with a distinct sulcus intermedius. Carpals poorly ossified, composed of radiale, ulnare, element Y, and large post-axial element formed by carpals 3–5. Carpal 2 has either been lost or fused to the latter element. Finger phalangeal formula is reduced (0-2-3-2), and the terminal phalanges of the second and fourth fingers are small, round elements. Prepollex absent.

Pubis unossified in ZSM 5943/2005 and fully ossified in ZSM 5942/2005; iliac shafts passing ventral to and beyond sacrum, oblong in cross-section, with a weak dorsal crest and without a dorsal prominence and with a shallow oblique groove. Femur weakly sigmoid, lacking a posterior crest. Tibiofibula equal to femur in length, with a sulcus intermedius. Tibiale and fibulare fused proximally and distally. T1 and T2+3 tarsals present, T1 considerably smaller than T2+3. Centrale present, slightly smaller than T2+3. Prehallux diminutive. Phalangeal formula reduced (1-2-3-4-3). Terminal phalanges of toes 3 and 4 with knobs, those of other toes small, round elements.

**Distribution, natural history, and conservation status.** This species is known only from Sainte Luce, southeast Madagascar (Fig 6). Records of ‘*Stumpffia tridactyla*’ from Mandena [47], and Vohimena mountains and the southern Anosy mountain chain [48], and of ‘*Stumpffia* sp. aff. *tetradactyla* “Southeast”‘ from Tsitongambarika [49] may refer to this species but require verification. A specimen from Nahampoana (ZFMK 53775) resembles this species, but due to the lack of genetic data, we cannot confirm its identity. Calls of *Stumpffia*-like frogs from Nahampoana were described in Glaw and Vences [50], but these were lower in dominant frequency (ca. 5 kHz), and longer in call duration (ca. 250 ms) than those recorded in Sainte Luce that are here assigned to *M*. *scule* sp. nov. Two separate ‘*Stumpffia*’ calls from Nahampoana were included in Vences et al. [51], one as Track 51, ‘*Stumpffia* sp. (Nahampoana)’, and a second as Cut 2 of Track 37, ‘*Stumpffia tetradactyla*’.

This species appears restricted to areas of deep leaf litter concomitant with semi-permanent water bodies such as shallow and slow-moving forest streams. Individuals call from concealed positions on adjacent stream banks during the day. Sainte Luce consists of 17 forest fragments (numbered S1–S17), covering approximately 1600 Ha of littoral forest. At present we assume that this species is microendemic to these forest fragments, and we have directly observed it in fragments S7, S8, and S9, but it appears to be absent from S1 and S2. It may also occur in other parcels of lowland forest nearby. Based on its current estimated Extent of Occurrence (= Area of Occupancy) of < 10 km^2^ in forest that is threatened and declining in quality despite protection status, we recommend this species be listed as Critically Endangered according to the IUCN Red List Criterion CR B1ab(iii) [44]. So far, no other described amphibian species are known to be restricted to Sainte Luce.

**Etymology.** We use the specific epithet ‘scule’ as an arbitrary combination of letters, in order to form a pun on ‘miniscule’ from the name in apposition, in reference to the fact that it is among the smallest known frogs from Madagascar and in the world. It is to be regarded as an invariable noun.

### *Mini ature* sp. nov.

urn:lsid:zoobank.org:act:0C1C4CE2-419A-4030-86D1-B1B89793697D

(Figs 1–3 and 9, Table 1)

**Remark.** This species was previously listed as *Stumpffia* sp. Ca53 MV2017(MF867231) by Tu et al. [20], though with incorrect accession number (correct number is MF768231).

**Holotype (Figs 2 and 9).** ZSM 86/2004 (FGZC 0151, GenBank accession numbers MF768231 and MK459307 for 5’ and 3’ fragments of the *16S rRNA* gene, respectively, and MF768147 for *cox1* gene), a presumed adult specimen collected in Andohahela National Park between Isaka and Eminiminy above ‘Camp 1’ (ca. 24.75°S, 46.85°E, ca. 350 m) between 29 and 31 January 2004 by F. Glaw, M. Puente, M. Teschke (née Thomas), and R.D. Randrianiaina.

**Diagnosis.** A highly miniaturised frog assigned to *Mini* gen. nov. on the basis of its small size, curved clavicles, laterally displaced and reduced nasals, and fusion or loss of carpal 2. This assignment is supported by its genetic affinities (Fig 1; [20]). It is separated by uncorrected p-distances of 10.0–10.6% in the analysed 3’ fragment of the *16S rRNA* gene from other members of the genus *Mini* gen. nov., and 10.8–13.7% from all members of the genus *Plethodontohyla*.

*Mini ature* sp. nov. is characterised by the unique combination of the following characters (n = 1 specimen): (1) SVL 14.9 mm; (2) ED/HL 0.38; (3) HW/SVL 37.2; (4) FARL/SVL 0.32; (5) TIBL/SVL 0.34; (6) HIL/SVL 1.18; (7) finger 1 strongly reduced, 2 and 4 reduced; (8) toe 1 absent, toes 2 and 5 quite reduced; (9) maxillary and premaxillary teeth present; (10) vomerine teeth absent; (11) lateral colour border absent; (12) black inguinal spots present; (13) postchoanal vomer present, spatulate, medially fused to parasphenoid; (14) nasal cultriform and laterally displaced; (15) quadratojugal-maxilla contact strong and broad; (16) zygomatic ramus of squamosal long, thin, curved, and horizontal; (17) clavicles present, curving with simple lateral articulations, medially not bulbous; (18) prepollex thin and cultriform; (19) carpal 2 absent or fused to post-axial carpal 3–5 element; (20) finger phalangeal formula 1-2-3-3; (21) toe phalangeal formula 1-2-3-4-3; (22–26) calls unknown.

Within the genus *Mini* gen. nov., the new species can be distinguished by its distinctly larger body size (14.9 mm vs 8.2–11.3 mm), shorter relative hindlimb length (HIL/SVL 1.18 vs 1.41–1.72) and phalangeal formula of fingers (1-2-3-3 vs 1-2-3-2 in *M*. *mum* sp. nov. and 0-2-3-2 in *M*. *scule* sp. nov.). Additionally, it can be distinguished from *M*. *mum* sp. nov. by the presence of maxillary and premaxillary teeth (vs absence), and less distinct lateral colour border, and *M*. *scule* sp. nov. by proportionally smaller nasals and braincase, broader quadratojugal-maxillary contact, and vertical dorsal process of premaxilla (vs anterior).

This species is particularly similar to some highly miniaturised *Stumpffia* species, but it can be distinguished from all *Stumpffia* based on the condition of the carpals and the presence of curved clavicles, and most *Stumpffia* by the presence of maxillary and premaxillary teeth.

**Holotype description.** Specimen in a moderately good state of preservation, the left arm removed as a tissue sample, the whole body somewhat dorsoventrally flattened in preservative. Body oblong; head wider than long, narrower than body width; snout slightly pointed in dorsal view, pointed in lateral view; nostrils directed laterally, not protuberant, equidistant between tip of snout and eye; canthus rostralis rounded, indistinct, slightly concave; loreal region flat, vertical; tympanum indistinct, round, ~48% of eye diameter; supratympanic fold absent; tongue long, broadening posteriorly, attached anteriorly, not notched; maxillary teeth present; vomerine teeth present; choanae small and round. Forelimb slender; subarticular tubercles single, elongated; outer/palmar metacarpal tubercle small and round; inner metacarpal tubercle slightly smaller than outer/palmar; hand without webbing; first finger strongly reduced, second and fourth fingers reduced, third finger basally broadened; relative lengths of fingers 1<2 = 4<3, fourth and second finger equal in length; finger tips not expanded into discs. Hindlimbs stocky; TIBL 34% of SVL; lateral metatarsalia strongly connected; inner metatarsal tubercle indistinguishable from first toe; outer metatarsal tubercle absent; no webbing between toes, second and fifth toes reduced; relative lengths of toes 2<5<3<4, fifth toe distinctly shorter than third; toe tips slightly pointed distally. Skin on dorsum smooth without a distinct dorsolateral fold, but with a distinct colour border, see below. Ventral skin smooth.

After 14 years in 70% ethanol, the dorsum is light brown, paler—almost beige—laterally, and slightly translucent, with a thin beige vertebral stripe and a darkened area on the posterior head. The skin above the eyes is translucent and dark in colour through the presence of the eyes beneath. The dorsal forelimb is beige flecked with brown, the hand is lighter medially, and the fingers have faint cream annuli. The dorsal hindlimb is beige in base colour with several brown crossbands on the thigh and shank that line up when the leg is folded together. A trapezoid of brown is present around the vent. The foot is dorsally as the forelimb with a light annulus before each distal phalanx. A distinct colour border that is not straight is present laterally, running along the canthus rostralis from the nostril through the eye, through the supratympanic region along the torso to the inguinal region. Small oblong dark brown spots are present in the inguinal region. The side of the head is dark brown. Ventral to this colour border the frog is mocha speckled with beige, lightening ventrally to beige with loose cream speckles. The ventral skin is translucent, and some of the organs can be seen through it. The chin is not differently coloured than the rest of the ventral body. This pattern continues onto the ventral limbs. No data on life colouration are available.

**Variation.** This species is currently known from a single specimen only.

**Osteology (Fig 9).** Based on ZSM 86/2004 (figured).

**Cranium (Fig 9D–9G).** Shape and proportions. Skull almost equilateral, roughly as wide as long, widest at quadratojugal-squamosal junction, lateral to the otic region. Braincase moderately broad, rostrum not shortened.

**Neurocranium.** Well ossified, including the otic capsules. The anterior cone of the sphenethmoid is ossified and contacts the frontoparietal dorsally but is not in contact with any other bones; small isolated lateral mineralisations of this bone are also present anterodorsal to the anterior tip of the cultriform process of the parasphenoid. Prootic in dorsal contact with lateral flange of frontoparietal, ventral contact with parasphenoid alae, not approaching contralateral ventrally. Septomaxilla miniscule, relatively poorly mineralised, and therefore not further discussed here. Columella (stapes) well ossified, pars media plectra (stylus) long and nearly straight, weakly posteriorly and dorsally oriented toward the reniform, dorsally elongated pars interna plectra (baseplate). Nasal narrow and cultriform, laterally displaced, curved downward laterally, acuminate maxillary process not closely approaching maxillary pars facialis, broadly separated from contralateral. Frontoparietal with rounded anterior edge, laterally bulging, with short lateral flange covering prootic, posteriorly strongly connected to exoccipital, anteroventrally contacting sphenethmoid, lacking any dorsal process.

Parasphenoid with narrow, rather straight-edged cultriform process and broad perpendicular alae, considerably shorter than frontoparietals, in contact with exoccipitals posterodorsally, prootics dorsally along the edges of the alae, anteroventrally in contact with postchoanal vomer and not in contact with neopalatine; posteromedial process excluded from participating in foramen magnum by exoccipitals. Vomer divided into pre- and postchoanal portions; prechoanal portion narrow, arcuate, triradiate, with a short lateral and anterior ramus and long, curving posterior ramus; postchoanal portion spatulate and edentate, contacting its contralateral on the midline, in dorsal contact with the parasphenoid proximally and the neopalatine distally, lacking an anterior projection. Neopalatine simple, straight, almost indistinguishable from lateral postchoanal portion of vomer, laterally broadly separated from the maxilla, not exceeding the lateral-most point of the postchoanal vomer.

Maxillary arcade fairly slight, maxilla and premaxilla bearing numerous diminutive teeth, anterior extension of maxilla exceeding lateral extent of premaxilla but not in contact with it. Premaxilla with a broad acuminate dorsal alary process rising laterally, pars palatina shallowly divided into a narrow palatine process and broad lateral process. Maxilla with a low triangular pars facialis and a narrow pars palatina, its posterior tip acuminate and broadly overlapping the quadratojugal, the lingual surface of the pars palatina running parallel to but not touching the anterior ramus of the pterygoid.

Pterygoid with a short medial ramus, long anterior ramus, and broad posterior ramus and posterolaterally calcified to the quadratojugal complex. Quadratojugal weakly bowed laterally, broadly connected to the ventral ramus of the squamosal, bearing a small posteroventral knob; the articulation of the mandible is apparently fortified by the mineralisation of the posterior ramus of the pterygoid+squamosal+quadratojugal posteroventral knob. Squamosal with a slender ventral ramus, broadened otic ramus, and long, thin zygomatic ramus, the otic ramus oriented slightly dorsally and posteriorly, the zygomatic ramus horizontal and strongly curved.

Mandible slim and edentate, largely unremarkable, with a low coronoid process on the angulosplenial. Mentomeckelians separated from the dentary, with slightly bulbous, almost hooked ventrolateral projections.

Posteromedial processes of hyoid proximally rounded with a broad medial crista.

**Postcranial skeleton (Fig 9A–9C, 9H and 9I).** Eight procoelous unfused presacrals, much broader than long, lacking neural spines, with round posterior articular processes, presacral I with a complete neural arch, presacrals II–IV with thicker and longer transverse processes than V–VIII. Sacrum with expanded diapophyses, the leading and trailing edges roughly equally angled, the articulation type IIB *sensu* Emerson [42]. Urostyle bicondylar, long, not broadening posteriorly, without lateral processes and with a low dorsal ridge.

Pectoral girdle without ossified prezonal or postzonal elements, with ossified clavicles. Clavicle thin and curved, with a simple lateral junction, shorter than the coracoid. Coracoid fairly narrow, not flared laterally, strongly flared medially with a large medial articular surface with the contralateral. Scapula slender, with a thin pars acromialis, the cleithral border concave. Cleithrum ossified for two thirds the width of the scapular border, thickened anteriorly. Suprascapula unossified.

Humerus with a well-developed crista ventralis and no medial or lateral cristae. Radioulna slender with a distinct sulcus intermedius. Carpals poorly ossified, composed of radiale, ulnare, prepollical element, element Y, and large post-axial element formed by carpals 3–5. Carpal 2 has either been lost or fused to the latter element. Finger phalangeal formula is reduced (1-2-3-3), and the terminal phalanx of the first finger is a small, round element. Small distal knobs on terminal phalanx of finger 3. Prepollex thin and cultriform and extending only to the base of the first metacarpal.

Pubis calcified, iliac shafts passing ventral to and beyond sacrum, nearly cylindrical, without a dorsal crest and with a weak dorsal prominence and shallow oblique groove. Femur weakly sigmoid, almost lacking a posterior crest. Tibiofibula shorter than femur, with a sulcus intermedius. Tibiale and fibulare fused proximally and distally. T1 and T2+3 tarsals present, T1 considerably smaller than T2+3, plus a small additional ossification (possibly a sesamoid) between the bases of metatarsals 2 and 3. Centrale present, roughly the size of T2+3. Prehallux subtriangular. Phalangeal formula reduced (1-2-3-4-3). Terminal phalanges of toes 2–4 with almost T-shaped knobs.

**Distribution, natural history, and conservation status.** This species is known only from a single specimen from Andohahela National Park in southeast Madagascar (Fig 6). The species is larger than the other members of the genus *Mini*, and its ecology may differ accordingly. Advertisement calls were not recorded. At present it is not possible to estimate its distribution or population status, and we prefer to suggest this species be considered Data Deficient until more information is available.

**Etymology.** We use the specific epithet ‘ature’ as an arbitrary combination of letters, in order to form a pun on ‘miniature’ from the name in apposition, in reference to the small size of this species. It is to be regarded as an invariable noun.

### *Rhombophryne proportionalis* sp. nov.

**Fig 10:**
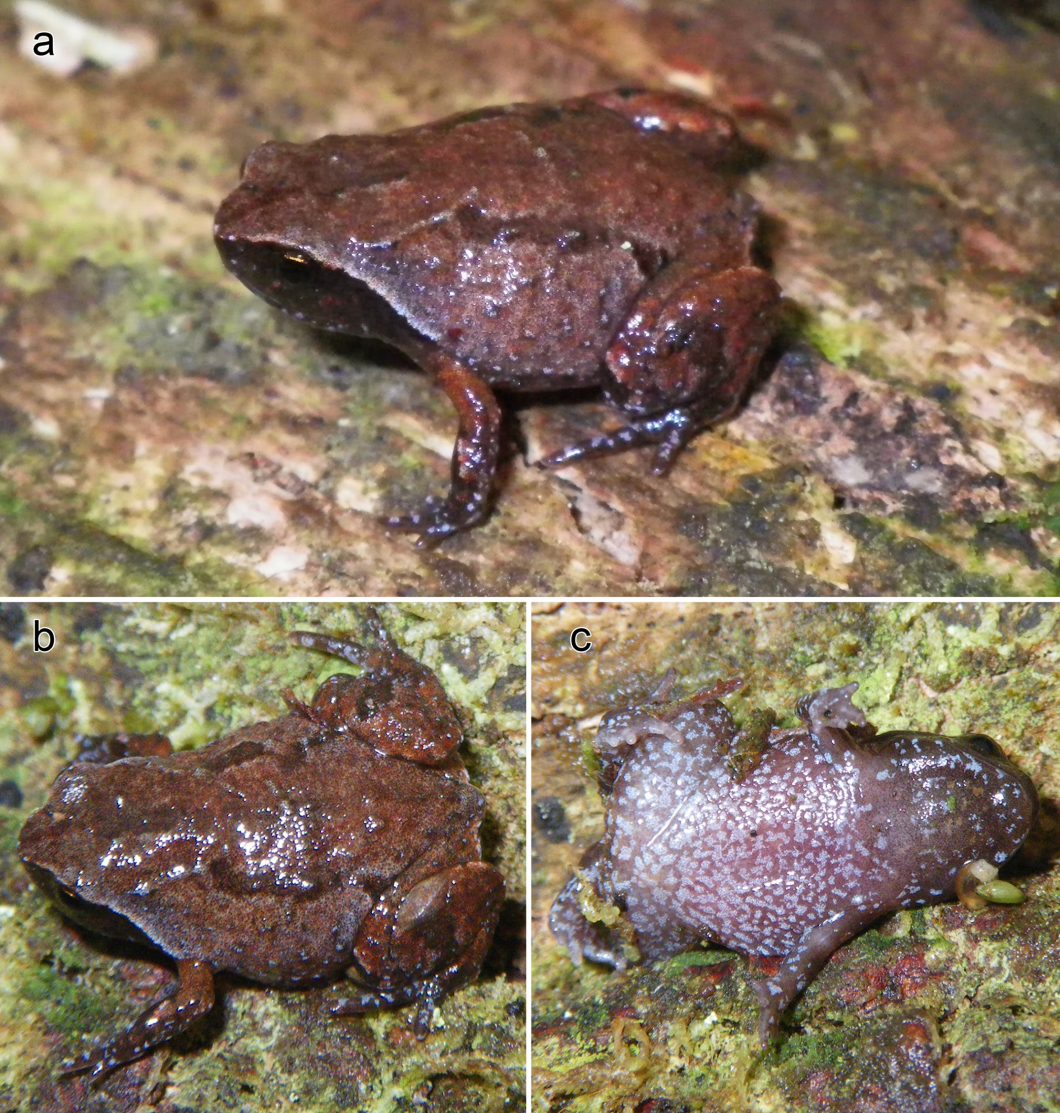
*Rhombophryne proportionalis* sp. nov., holotype ZSM 1826/2010, in life. (a) dorsolateral, (b) dorsal, and (c) ventral view.

**Fig 11:**
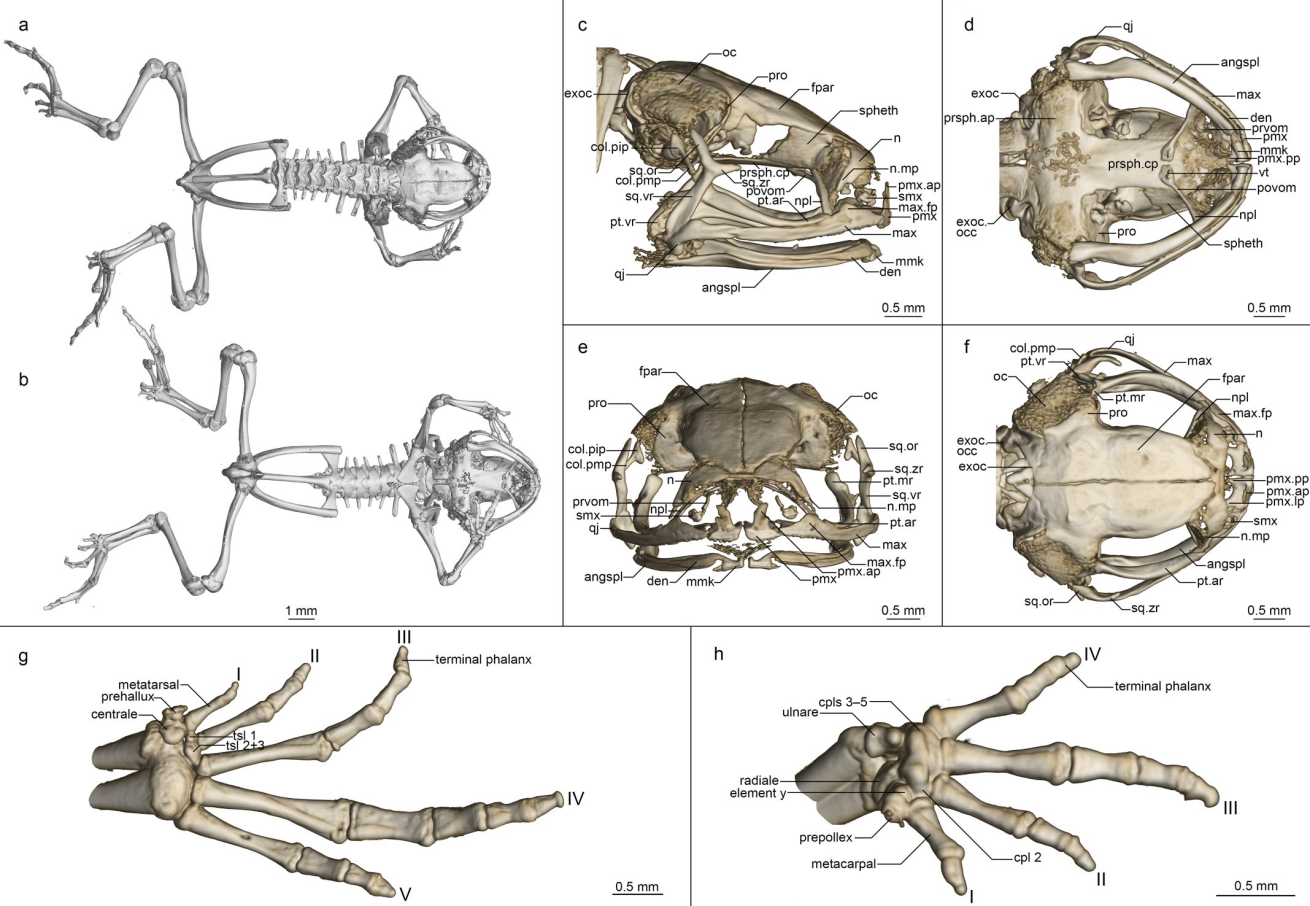
Osteology of *Rhombophryne proportionalis* sp. nov. holotype (ZSM 1826/2010). (a, b) Whole skeleton in (a) dorsal and (b) ventral view. (c-f) Skull in (c) lateral, (d) ventral, (e) anterior, and (f) dorsal view. (g) Foot in ventral view. (h) Hand in ventral view. For abbreviations, see Fig 6.

urn:lsid:zoobank.org:act:1A79607B-E9D4-4214-BF47-9BC0B4320E5F

(Figs 1, 2, 3, 5, 10 and 11, Tables 1 and 2)

**Remark.** This species was previously referred to as *Stumpffia* sp. Ca34 [15, 46] and as *Stumpffia* sp. 39 MV-2012 (KC351481) [20].

**Holotype.** ZSM 1826/2010 (ZCMV 12404, GenBank accession number KC351380 and KU937808 for 5’ and 3’ fragments of the *16S rRNA* gene, respectively, and KF611640 for the *cox1* gene), an adult male specimen (seen calling, not recorded) collected at Bepia Campsite on the Tsaratanana massif (Camp 3; 14.1182°S, 48.9782°E, 2294 m a.s.l.), Diana Region, former Antsiranana province, northern Madagascar on 16 June 2010 by M. Vences, D.R. Vieites, R.D. Randrianiaina, S. Rasamison, and E. Rajeriarison.

**Paratypes.** ZSM 1840/2010 (ZCMV 12405, GenBank accession number KC351481 and MK459317 for 5’ and 3’ fragments of the *16S rRNA* gene, respectively), adult male specimen with the same collection data as the holotype; and ZSM 636/2014 (DRV 6224), an adult presumed male specimen collected at Andranomadio Campsite in Tsaratanana (Camp 4; 14.0801°S, 48.9854°E, 2503 m a.s.l.) on 16 June 2010 by the same collectors.

**Diagnosis.** A diminutive frog assigned to the genus *Rhombophryne* on the basis of absence of clavicles, presence of vomerine, maxillary, and premaxillary teeth, short and broad skull, and genetic affinities. It is separated by uncorrected p-distances of 7.0–12.9% in the analysed 3’ fragment of the *16S rRNA* gene from other members of the genus *Rhombophryne*.

*Rhombophryne proportionalis* sp. nov. is characterised by the unique combination of the following characters (n = 3 male specimens): (1) male SVL 11.0–12.3 mm; (2) ED/HL 0.40–0.48; (3) HW/SVL 0.33–0.37; (4) FARL/SVL 0.33–0.35; (5) TIBL/SVL 0.34–0.36; (6) HIL/SVL 0.21–1.32; (7) finger 1 reduced, 2 and 4 short; (8) toe 1 highly reduced, 2 somewhat reduced; (9) maxillary and premaxillary teeth present; (10) vomerine teeth present; (11) lateral colour border absent; (12) black inguinal spots sometimes present; (13) postchoanal vomer present, spatulate, medially fused to parasphenoid; (14) nasal broad and not laterally displaced; (15) quadratojugal-maxilla contact strong and broad; (16) zygomatic ramus of squamosal long, thick, curved, and horizontal; (17) clavicles absent; (18) prepollex short and triangular; (19) carpal 2 present; (20) finger phalangeal formula 2-2-3-3; (21) toe phalangeal formula 2-2-3-4-3; (22) unpulsed calls emitted in series of 9–17 calls at irregular intervals; (23) non-frequency modulated calls; (24) call dominant frequency 5460 ± 117 Hz (n = 79); (25) call duration 45.4 ± 8.2 ms (n = 79); (26) inter-call interval 63.0 ± 9.0 ms (n = 73).

Among extremely miniaturized cophylines, this species is unique in possessing vomerine teeth. It can be distinguished from almost all other miniaturised species in lacking clavicles (also absent in *S*. *contumelia*, *S*. *obscoena*, *S*. *davidattenboroughi*, *S*. *makira*, *S*. *achillei*, and *S*. *analanjirofo*, and some specimens of *S*. *tridactyla*, unpublished data). It is also characterised by a dark colouration of the lateral surface of the head with a distinct colour border, and less reduced fingers and toes. Confusion with juvenile *Rhombophryne* species is still possible, but these have much larger teeth proportional to their skull size, and most lack the distinct lateral head colouration and possess clavicles. The call is unique among the frogs of Madagascar and is instantly distinctive in being emitted as a rapid, high-pitched series of tonal notes.

**Holotype description.** Specimen in a good state of preservation, part of the right thigh removed as a tissue sample. Body somewhat rhomboid; head wider than long, narrower than body; snout rounded in dorsal view, squared in lateral view; nostrils directed laterally, not protuberant, closer to eye than to tip of snout; canthus rostralis rounded, concave; loreal region flat, vertical; tympanum indistinct, round, about 57% of eye diameter; supratympanic fold distinct, weakly raised, curving slightly from posterior corner of eye over tympanum toward axilla; tongue very broad, disc-like, posteriorly free, unlobed; maxillary teeth present; vomerine teeth present in two tiny patches either side of the midline; choanae rounded. Forelimbs slender; subarticular tubercles faint, single; outer metacarpal tubercle faint, paired; inner metacarpal tubercle distinct, elongated; hand without webbing; first finger reduced, second and fourth short; relative length of fingers 1<2 = 4<3; finger tips not expanded. Hind limbs robust; TIBL 34% of SVL; lateral metatarsalia strongly connected; inner metatarsal tubercle thin and indistinct; outer metatarsal tubercle small and indistinct; no webbing between toes; first toe highly reduced, second toe short; relative length of toes 1<2<5<3<4; fifth toe distinctly shorter than third. Skin on dorsum smooth in preservative, without distinct dorsolateral folds. In life, the dorsal skin was smooth was scattered tubercles, and distinct ridges above the scapular region (Fig 10). Ventral skin smooth in preservative, granular in life.

After eight years in 70% ethanol, the dorsum is chocolate brown in colour, darker over the head, with a faint dark brown line running from the inguinal region anteriorly toward the eye. The dorsal leg has a dark brown crossband on the shank. The lateral head has a distinct colour border to the dorsum, being a much darker brown, its border defined by the supratympanic fold. The flank has an indistinct colour border to the venter. The venter is brown with cream flecks, slightly darker and less flecked on the chin. The ventral legs and arms are as the ventral abdomen in colour. The bottom of the foot is dark brown along the medial half. Colour in life as in preservative but more vibrant.

**Variation.** For measurements, see Table 1. The paratypes are in general very similar to the holotype in morphology. ZSM 636/2014 is slightly smaller than the other specimens, and has a slightly shorter, more rounded head. Its supratympanic fold is also less curved than those of the other specimens, being rather more straight from the eye to above the arm. ZSM 1840/2010 has a more massive body than the others. The colouration of the specimens is relatively consistent, with the whole venter of ZSM 1840/2010 being darker than those of the other two specimens. The dorsolateral lines of the holotype are present in ZSM 1840/2010, but not in ZSM 636/2014, instead being broken in that specimen into spots above the suprascapular region and lines in the inguinal region. The crossbands of the shanks are less distinct in ZSM 1840/2010 than the other two specimens.

**Bioacoustics.** Specimens called only during the day, from open, shrubby landscape between dense vegetation, on the ground. In some areas, numerous specimens could be heard calling in a chorus. Calls were recorded from an uncollected specimen by M. Vences (Fig 5C, Table 2) at around 11h40 on 15 June 2010 in Camp Bepia (14.11822°S, 048.97822°E, 2294 m a.s.l.), and further calls were heard but not recorded at Camp Andranomadio (14.0801°S, 048.9854°E, 2503 m a.s.l.). A precise description of the call structure is difficult; the vocalization is a series of short tonal units that cannot readily be assigned to units. In past studies, we have described roughly comparable structures differently: in *Stumpffia psologlossa*, where the units have a very short duration, as a single call composed of pulses [14], in *Rhombophryne mangabensis* as a series of notes [52], and in *R*. *minuta* two closely spaced units were seen as components of a single note [52]. To allow comparison within *Rhombophryne* we here define the tonal units in the vocalizations of the new species as notes, and the entire series as a call, but emphasize that it also would be possible to define each unit as call and the entire series as call series, or to consider each unit as a pulse as their duration falls within the 5–50 ms range for which the pulse category was recommended by Köhler et al. [33].

Calls are rapid series of high-pitched slightly frequency modulated notes. Each call consists of a series of 9–17 notes, and calls are repeated at long and irregular intervals. For detailed call parameters, see Table 2.

**Osteology (Fig 11).** Based on ZSM 1826/2010 (figured) and ZSM 636/2014 (not figured).

**Cranium (Fig 11C–11F).** Shape and proportions. Skull short and stout, relatively wide, widest at quadratojugal anterior to the otic region. Short rostrum. Braincase broad.

**Neurocranium.** Well ossified except the weakly calcified otic capsules. Sphenethmoid well ossified, in contact with the frontoparietal in ZSM 1826/2010 but not in ZSM 636/2014, with a calcified anterior medial cone; broadly separated from prootic. Prootic in dorsal contact with lateral flange of frontoparietal, ventral contact with parasphenoid alae, not approaching contralateral. Septomaxilla miniscule, roughly spiralled, similar in shape to those of other *Rhombophryne* (see [25]). Columella (stapes) well ossified, pars media plectra (stylus) steeply sloping upwards proximally to its broad, bilobed, flattened pars interna plectra (baseplate). Nasal broad, curved over the nasal capsule, triangular, acuminate maxillary process not closely approaching maxillary pars facialis, broadly separated from contralateral, situated anterior to frontoparietal. Frontoparietal with slanted anterior edge, laterally bulging, with short lateral flange covering prootic, posteriorly weakly connected to exoccipital, anteroventrally contacting sphenethmoid, lacking any dorsal process (suggestions of such processes in ZSM 1826/2010).

Parasphenoid with broad, squared cultriform process and broad perpendicular alae, shorter than frontoparietals, posteromedial process not participating in foramen magnum, anteroventrally in contact with postchoanal vomer and not in contact with neopalatine. Vomer divided into pre- and postchoanal portions; prechoanal portion narrow, arcuate, triradiate, with a short lateral and anterior ramus and long posterior ramus; postchoanal portion spatulate bearing a single vomerine tooth or a pair thereof, separated from contralateral by a narrow space, in dorsal contact with the parasphenoid proximally and the neopalatine distally, lacking an anterior projection. Neopalatine laminar, broader than lateral postchoanal portion of vomer, laterally approaching but not contacting the maxilla, exceeding the lateral-most point of the postchoanal vomer to approach the anterior tip of the parasphenoid but not in contact with it, such that the vomer contacts the neopalatine near its midpoint and not at its terminus.

Maxillary arcade quite robust, maxilla and premaxilla bearing numerous diminutive teeth, premaxilla and maxilla in narrow contact anteriorly. Premaxilla with a broad acuminate dorsal alary process rising vertically, pars palatina shallowly divided into a narrow palatine process and broad lateral process. Maxilla with a low triangular pars facialis and a narrow pars palatina, its posterior tip acuminate and broadly overlapping the quadratojugal, the lingual surface of the pars palatina in broad contact with the anterior ramus of the pterygoid.

Pterygoid with a short medial ramus, long anterior ramus with broad contact with the maxilla, leaving a channel for the pterygoid cartilage, posterior ramus broad and posterolaterally calcified to the quadratojugal complex. Quadratojugal bowed laterally, broadly connected to the ventral ramus of the squamosal, and with a broad articular surface with the maxilla, bearing a large posteroventral knob. Squamosal with a broadened ventral ramus and narrow otic and zygomatic rami, the otic ramus long and thin and oriented dorsally, the zygomatic ramus short and thin, oriented anteriorly.

Mandible slim and edentate, largely unremarkable, with a low coronoid process on the angulosplenial. Mentomeckelians strongly connected to the dentary, with unusual, flat ventrolateral projections.

Posteromedial processes of hyoid proximally pointed with a broad medial crista.

**Postcranial skeleton (Fig 11A, 11B, 11G and 11H).** Eight procoelous unfused presacrals, much broader than long, lacking neural spines, with round posterior articular processes, presacral I with a mostly complete neural arch, presacrals II–IV with thicker and longer transverse processes than V–VIII. Sacrum with expanded diapophyses, the leading and trailing edges roughly equally angled, the articulation type IIB *sensu* Emerson [42]. Urostyle bicondylar, long, broadening posteriorly, without lateral processes and with a low dorsal ridge.

Pectoral girdle without ossified prezonal or postzonal elements, lacking ossified clavicles. Coracoid broad, strongly flared with a large medial articular surface with the contralateral. Scapula also robust, with a broad pars acromialis distinct from the pars glenoidalis. Cleithrum ossified for half the width of the scapular border, acuminate, thickened anteriorly. Suprascapula unossified.

Humerus with a well-developed crista ventralis and no medial or lateral cristae. Radioulna broad with a distinct sulcus intermedius. Carpals well ossified in ZSM 1826/2010 and poorly ossified in ZSM 636/2014, composed of radiale, ulnare, prepollical element, element Y, carpal 2, and large post-axial element formed by carpals 3–5. Finger phalangeal formula is standard (2-2-3-3). Small distal knobs on terminal phalanges of the fingers. A very small prepollex is present in ZSM 1826/2010 but is not visible in ZSM 636/2014.

Pubis partly calcified, iliac shafts passing ventral to and beyond sacrum, nearly cylindrical, without a dorsal crest and with a weak dorsal prominence and shallow oblique groove. Femur weakly sigmoid with a low posterior crest. Tibiofibula shorter than femur, with a sulcus intermedius. Tibiale and fibulare weakly fused proximally and distally. T1 and T2+3 tarsals present, T1 considerably smaller than T2+3. Centrale present but not large. Prehallux unossified. Phalangeal formula standard (2-2-3-4-3).

**Distribution, natural history, and conservation status.** *Rhombophryne proportionalis* sp. nov. is known from two localities (Bepia and Andranomadio) on the Tsaratanana massif in northern Madagascar. Both of these sites fall within the Tsaratanana National Park (formerly a Strict Nature Reserve). It is a terrestrial species that lives among the leaf litter. Two other cophyline frogs, *Platypelis alticola* and *P*. *tsaratananaensis*, are also known from these locations, and both are currently listed as Endangered, due to their small distribution < 2000 km^2^, presence in a single threat-defined location, and potentially on-going decline in habitat quality. We therefore suggest *R*. *proportionalis* sp. nov. also to be Endangered, following the same rationale.

**Etymology.** The species epithet is the Latin adjective *proportionalis* meaning ‘proportional’, in reference to the comparatively proportional dwarfism that this species has apparently undergone (see discussion). It is a feminine adjective in the nominative singular.

### *Anodonthyla eximia* sp. nov.

**Fig 12:**
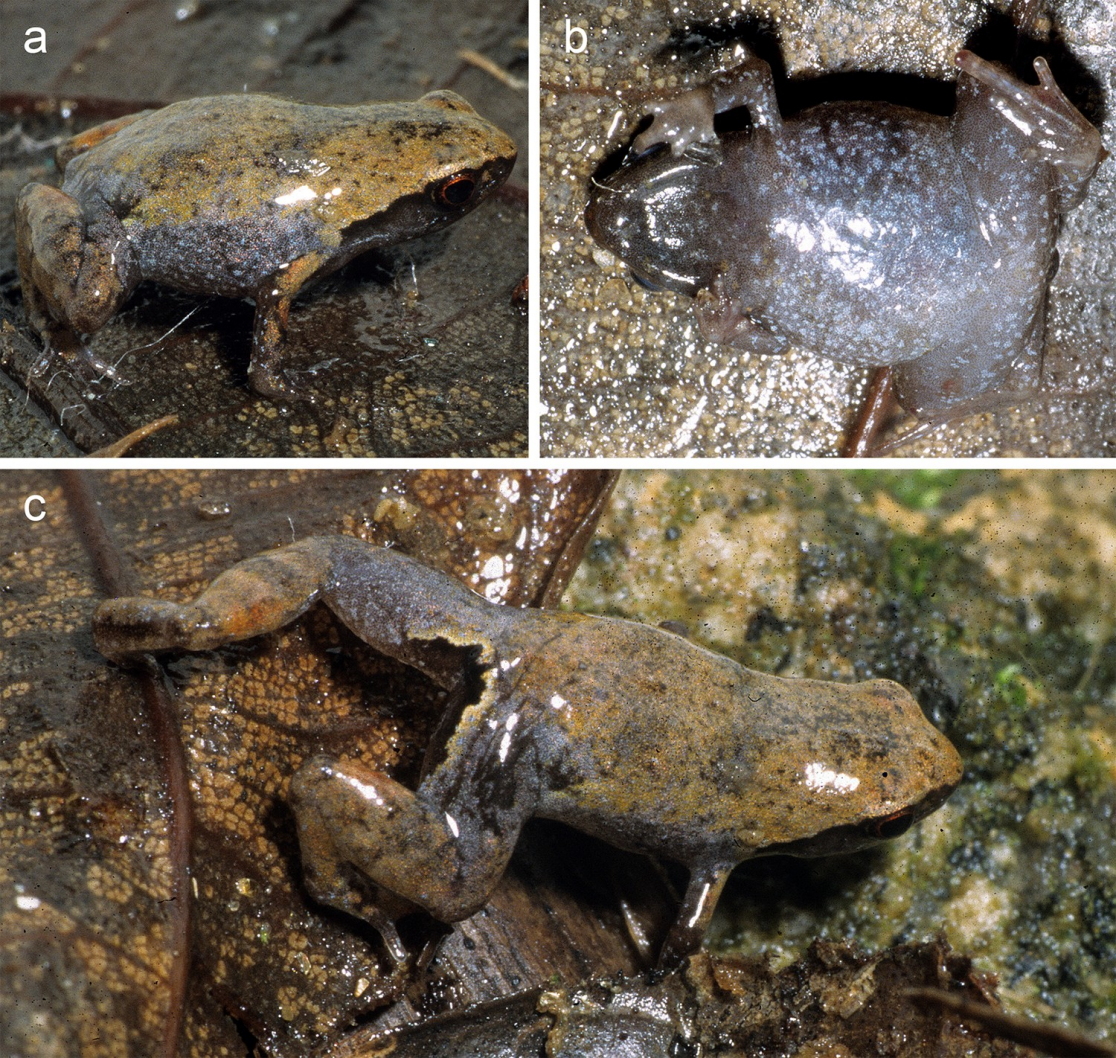
*Anodonthyla eximia* sp. nov. holotype (ZMA 20246) in life. (a) dorsolateral, (b) ventral, and (c) posterodorsal view.

**Fig 13:**
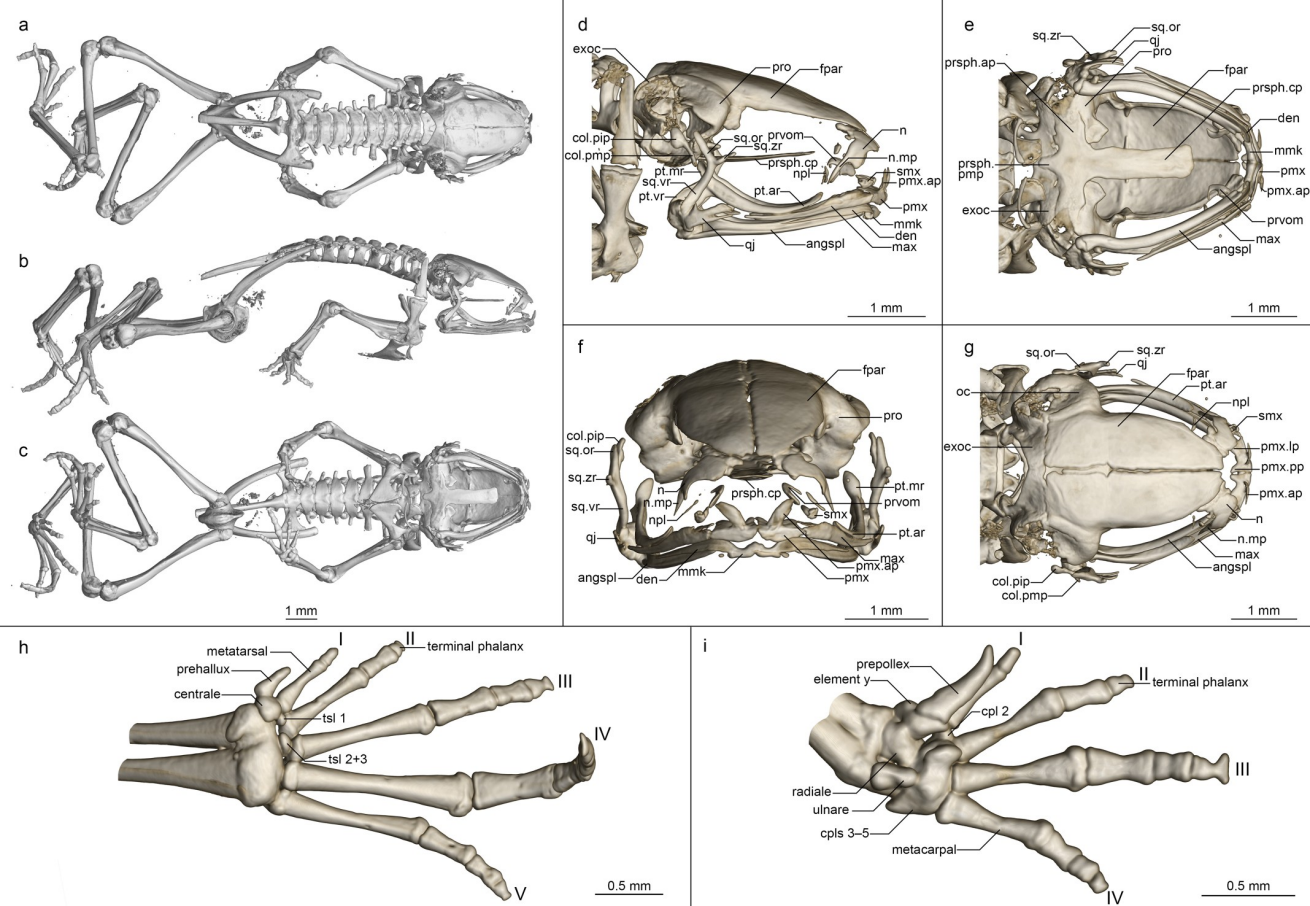
Osteology of *Anodonthyla eximia* sp. nov. holotype (ZMA 20246). (a-c) Whole skeleton in (a) dorsal, (b) lateral, and (c) ventral view. (d-g) Skull in (d) lateral, (e) ventral, (f) anterior, and (g) dorsal view. (h) Foot in ventral view. (i) Hand in ventral view. For abbreviations, see Fig 6.

urn:lsid:zoobank.org:act:2C419E74-C13D-447D-BDCF-312324515EAE

(Figs 1, 2, 3, 5, 12 and 13, Tables 1 and 2)

**Remark.** This species was previously listed as *A*. sp. Ranomafana (Maharira) [24], *A*. sp. Ca04 Ranomafana (ZCMV204) [15] and *Anodonthyla* sp. 4 Ranomafana [20].

**Holotype.** ZMA 20246 (ZCMV 204, GenBank accession number GU177052 and FJ559111 for the 5’ and 3’ fragments of the *16S rRNA* gene, respectively, and GU177063 for the *cox1* gene), an adult male specimen (vocal sac inflated when collected) collected from a campsite at the base of Maharira mountain (21.3258°S, 47.4025°E, approx. 1248 m a.s.l.) in Ranomafana National Park, Vatovavy-Fitovinany Region, former Fianarantsoa province, southeastern Madagascar on 25 January 2004 by M. Vences, I. de la Riva, and E. Rajeriarison.

**Diagnosis.** An extremely miniaturised frog assigned to the genus *Anodonthyla* on the basis of the possession of a large, cultriform prepollex, T-shaped terminal phalanges, absence of postchoanal vomer, and by its genetic affinities. It is separated by uncorrected p-distances of 9.3–17.0% in the analysed 3’ fragment of the *16S rRNA* gene from all other members of the genus *Anodonthyla*.

*Anodonthyla eximia* sp. nov. is characterised by the unique combination of the following characters (n = 1 male specimen): (1) male SVL 11.3 mm; (2) ED/HL 0.45; (3) HW/SVL 0.31; (4) FARL/SVL 0.30; (5) TIBL/SVL 0.39; (6) HIL/SVL 1.34; (7) finger 1 highly reduced, other fingers small; (8) toe 1 absent, toes 2 and 5 quite reduced; (9) maxillary and premaxillary teeth absent; (10) vomerine teeth absent; (11) lateral colour border absent; (12) black inguinal spots absent; (13) postchoanal vomer absent; (14) nasal subrectangular with an acuminate maxillary process, not displaced; (15) quadratojugal-maxilla contact absent; (16) zygomatic ramus of squamosal short, thin, and anterodorsally oriented; (17) clavicles present, curving with simple lateral articulations, medially bulbous; (18) prepollex cultriform, longer than first metatarsal; (19) carpal 2 present; (20) finger phalangeal formula 1-2-3-3; (21) toe phalangeal formula 2-2-3-4-3; (22) single-note, unpulsed calls, not emitted in series; (23) weakly frequency modulated calls; (24) call dominant frequency 8406 ± 78 Hz (n = 5); (25) call duration 59.6 ± 6.5 ms (n = 5); (26) inter-call interval 3749.0 ± 1149.9 ms (n = 4).

This species is considerably smaller than all other *Anodonthyla* species (11.3 mm vs 15–34 mm), and as such is only possible to confuse with juveniles of its congeners. In addition to its small size, it can be distinguished from all other *Anodonthyla* species by the absence of flared crests on the humerus in adult males. Among similarly sized adult frogs in Madagascar, it can be distinguished by the possession of a large inner metacarpal tubercle with a large, cultriform prepollex (present only in male *Anodonthyla*, much smaller in all other species except *Anilany*, where it is broad and triangular instead of cultriform), and a laterally displaced neopalatine not in contact with the sphenethmoid or vomer (neopalatine either in contact with sphenethmoid or in contact with vomer in all other species; in some species of *Stumpffia*, the neopalatine is lost, unpublished data).

**Holotype description.** Specimen in a good state of preservation, a piece of the right thigh removed as a tissue sample. Body oblong; head wider than long, narrower than the body width; snout rounded in dorsal and lateral view; nostrils directed laterally, not protuberant, closer to eye than to tip of snout; canthus rostralis rounded, straight; loreal region concave, vertical; tympanum indistinct, round, about 46% of eye diameter; supratympanic fold weak, not raised, straight from posterior corner of eye to axilla; tongue long, thin, attached anteriorly, not notched; maxillary teeth absent, vomerine teeth absent; choanae oblong, very small. Forelimbs relatively broad; subarticular tubercles indistinct, single; outer metacarpal tubercle small, indistinct, single; inner metacarpal tubercle large and distinct, bulging outward strongly, underlain by cultriform prepollex; hand without webbing; all fingers short, first highly reduced and only marginally longer than the prepollex; relative length of fingers 1<2 = 4<3, fourth finger equal in length to second; tip of third finger marginally expanded, other fingers not expanded into discs. Hind limbs robust; TIBL 39% of SVL; lateral metatarsalia strongly connected; inner metatarsal tubercle rounded, indistinguishable from first toe; outer metatarsal tubercle absent; no webbing between toes; first toe practically absent, second and fifth toes shortened; relative length of toes 2<5<3<4; fifth toe distinctly shorter than third. Skin on dorsum smooth, without distinct dorsolateral folds. Ventral skin smooth.

After 13 years in 70% ethanol, the dorsum is pale brown with a faint darker brown chevron over the scapular region (Fig 2). The dorsal legs have faint dark brown crossbands, especially on the shanks. The dorsal colouration fades over the shanks to cream, which is continuous on the venter. The lateral head is dark brown, with a distinct border to the dorsum and flank formed by the supratympanic fold. The chin is a lighter brown than the dorsum but distinctly not the cream of the abdomen. The ventral legs are translucent cream. Colour in life as in preservative but more vibrant; the venter was slate grey with blue-cream flecks.

**Bioacoustics.** Calls recorded from an unknown specimen by M. Vences at 08h45, on 26 January 2004 at Maharira in Ranomafana National Park (21.3258°S, 047.4025°E, 1248 m a.s.l.) in the leaf litter of primary rainforest, referred to this species (Fig 4D, Table 2). The holotype and single known specimen of this species was collected during careful searches of the leaf litter from the exact spot where similar calls were heard, and upon capture, had an apparent partially inflated vocal sac. No other calls assignable to a miniature frog were heard in the area at this time in the morning. Assignment of the calls to this species therefore is the most likely hypothesis but remains tentative. The call bears remarkable resemblance to that of *Stumpffia miery* (Table 2), which is also known from lower elevations in Ranomafana National Park, and detailed future field study will be required to confirm its assignment to *Anodonthyla eximia* sp. nov.

Calls were emitted as part of a large chorus in the early hours of the morning following cyclonic rainfall after the retreat of major flooding of the area. Calls consisted of a single note and were emitted at regular intervals without defined call series. Calls were weakly frequency modulated with an increase in pitch, but recording quality is too poor for detailed analysis. For approximate call parameters, see Table 2.

**Osteology (Fig 13).** Based on ZMA 20246 (figured).

**Cranium (Fig 13D–13G)**. Shape and proportions. Skull short and rounded, longer than wide, widest at the bowing of the quadratojugal roughly in line with the anterior face of the prootic. Braincase proportionally broad, with an extremely short rostrum.

**Neurocranium.** Moderately ossified, otic capsules partly ossified. Sphenethmoid unossified. Prootic in dorsal contact with lateral flange of frontoparietal, ventral contact with parasphenoid alae, not approaching contralateral. Septomaxilla miniscule, very tightly curled, not further discussed due to low ossification and insufficient resolution. Columella (stapes) well ossified, pars media plectra (stylus) long and slightly curved, posteriorly and dorsally oriented toward the broadened, somewhat posteriorly oriented pars interna plectra (baseplate). Nasal narrow, retaining the shape of larger cophylines: subrectangular with an elongated, acuminate maxillary process that does not closely approach the maxillary pars facialis; displaced laterally, broadly separated from contralateral. Frontoparietal with rounded anterior edge, laterally rather straight-edged, with short lateral flange covering prootic, posteriorly strongly connected to exoccipital, lacking any dorsal process, separated from contralateral by a narrow gap with a small rhomboid facet at the level of the prootics, which may represent a pineal foramen.

Parasphenoid with narrow, rather straight-edged cultriform process and roughly equally broad perpendicular alae, considerably shorter than frontoparietals, in contact with exoccipitals posterodorsally, prootics dorsally along the edges of the alae, anteroventrally free; posteromedial process long but not participating in foramen magnum. Vomer lacking postchoanal portion (typical of *Anodonthyla*); prechoanal portion strongly curved, bearing a small lateral ramus. Neopalatine simple and very thin, laterally displaced, not contacting any other ossified elements.

Maxillary arcade gracile, maxilla and premaxilla edentate, anterior extension of maxilla not exceeding lateral extent of premaxilla. Premaxilla with a narrow dorsal alary process rising laterally, pars palatina shallowly divided into a narrow palatine process and broad lateral process. Maxilla practically lacking a pars facialis and bearing a narrow pars palatina, its posterior tip acuminate and not contacting the quadratojugal, the lingual surface of the pars palatina contacting the anterior ramus of the pterygoid, which has taken over the articulation. Pterygoid with a short medial ramus, long and strongly curved anterior ramus, and broad posterior ramus, posteriorly calcified to the quadratojugal complex. Quadratojugal bowed laterally, rather short, broadly connected to the ventral ramus of the squamosal, bearing a small posteroventral knob, anteriorly dividing and with decreasing ossification, not connected to the maxilla; the articulation of the mandible is apparently somewhat fortified by the mineralisation of the posterior ramus of the pterygoid+squamosal+quadratojugal posteroventral knob. Squamosal with a slender, rather straight ventral ramus, thin, posterodorsally oriented otic ramus, and short, thin, anterodorsally oriented zygomatic ramus.

Mandible slim and edentate, largely unremarkable, with a weakly raised coronoid process on the angulosplenial. Mentomeckelians separated from the dentary, with small, poorly ossified hooked ventrolateral projections.

Posteromedial processes of hyoid proximally rounded without an obvious medial crista.

**Postcranial skeleton (Fig 13A–13C, 13H and 13I).** Eight procoelous presacrals, all much broader than long, lacking neural spines, with round posterior articular processes, presacral I with a complete neural arch, presacrals II–IV with thicker and longer transverse processes than V–VIII. Sacrum with flared diapophyses, the leading and trailing edges roughly equally curved, the articulation type IIB *sensu* Emerson [42]. Urostyle bicondylar, long, broadening gently posteriorly, with a somewhat flared head and a low dorsal ridge.

Pectoral girdle without ossified prezonal or postzonal elements, with ossified clavicles. Clavicle thin and curved, with a simple lateral junction, equal in length to the coracoid, medially bulbous. Coracoid broad, flared laterally and strongly flared medially with a curving medial articular surface with the contralateral. Scapula robust, with a broad pars acromialis, the cleithral border straight. Cleithrum ossified for three-quarters the width of the scapular border, thickened anteriorly. Suprascapula unossified.

Humerus with a well-developed crista ventralis and no medial or lateral cristae. Radioulna slender with a distinct sulcus intermedius. Carpals composed of radiale, ulnare, element Y, prepollex, carpal 2, and a large post-axial element formed by carpals 3–5. Prepollex extremely long and acuminate, longer than first metacarpal. Finger phalangeal formula is reduced (1-2-3-3), and the terminal phalanx of the first finger small and columnar, others bearing T-shaped knobs.

Pubis ossified; iliac shafts passing ventral to and beyond sacrum, oblong in cross-section, with a weak dorsal crest, a distinct dorsal prominence, and a shallow oblique groove. Femur weakly sigmoid, bearing a distinct posterior crest. Tibiofibula slightly shorter than femur in length, with a sulcus intermedius. Tibiale and fibulare fused proximally and distally. T1 and T2+3 tarsals present, T1 considerably smaller than T2+3. Centrale present, larger than other tarsals. Prehallux elongated, half the length of first metatarsal. Phalangeal formula standard (2-2-3-4-3). Terminal phalanx of toe 1 a small round element, those of toes 2–5 with T-shaped knobs.

**Etymology.** The species epithet *eximia* is the feminine form of the Latin adjective *eximius* meaning ‘remarkable’ or ‘special’, in reference to the surprisingly small body size and terrestrial habits of this *Anodonthyla* species.

**Distribution, natural history, and conservation status.** *Anodonthyla eximia* sp. nov. is known only from Maharira in Ranomafana National Park (Fig 6). It is a terrestrial species. Nothing else is known of its natural history. It is likely that this species should be classified as Vulnerable like other species from Maharira (e.g. *Gephyromantis runewsweeki*), but as we know almost nothing of its range and ecology, we instead recommend that it be considered Data Deficient until more data are available.

## Discussion

### Genus-level taxonomy of the Cophylinae

*Mini* adds a ninth genus to the Cophylinae for a unique clade of miniaturised frogs that falls sister to the large-bodied *Plethodontohyla* (Fig 1). Although body size is the most obvious character that differentiates these two sister genera, they are also distinguished by a number of osteological features, and can be identified without skeletal analysis by their digital reduction (present in *Mini*, absent in *Plethodontohyla*) and vomerine teeth (absent in *Mini*, present in *Plethodontohyla*). Despite these differences and consistent recovery of the two genera as being reciprocally monophyletic in genetic analyses, the uncorrected p-distances between these genera in the 3’ fragment of the *16S rRNA* mitochondrial gene analysed here are at first glance surprisingly small at 8.3–13.3% (these distances would be distinctly higher if insertions and deletions would be considered in their calculation). Nevertheless, we consider the differences between these clades sufficiently great and robust that we regard them as constituting separate genera. Aside from their morphological and osteological differentiation, a further argument for their classification in distinct genera comes from the strength of support for their sister-group relationship; while the two clades here seen as genera *Plethodontohyla* and *Mini* have been placed sister to each other in most molecular analyses so far, support values for this grouping often were low, and typically lower than the respective support for each of the two clades. The clade stability criterion [36] is therefore better served considering both clades as separate genera.

The relationship of *Mini* to *Plethodontohyla* is analogous to the relationship of *Stumpffia* to *Rhombophryne*: a genus-level sister clade of miniaturised frogs (although *Stumpffia* also contains several non-miniaturised species), recovered in robust genetic phylogenies as reciprocally monophyletic, and distinguished by several diagnostic characters [14, 15, 23]. Peloso et al. [19] argued for the lumping of *Stumpffia*, *Rhombophryne*, and later also *Anilany* [22] into a single genus, *Rhombophryne*. In response, we showed that the initial argument for lumping was based on misidentified specimens [15], and subsequently incorrectly coded morphology, ignoring various unique diagnostic features of *Anilany*, and the relationships of particularly unstable taxa (most notably *Stumpffia tridactyla*) [23]. We revised the taxonomy of the genus *Stumpffia*, describing 26 new species, and providing a more robust phylogeny that resolved the phylogenetic position of *Stumpffia tridactyla* [14]. Despite this progress, the Amphibian Species of the World database (ASW) currently continues to use the lumped taxonomy, in contrast to AmphibiaWeb and other researchers that have adopted our proposed taxonomy (e.g. [20, 53]). The newly described *Rhombophryne proportionalis*, which is the only miniaturised *Rhombophryne* so far known, is highly distinct from *Stumpffia*, lacking, for example, the externally obvious digital reduction that is present in all miniaturised species of *Stumpffia*, and differing in body shape and proportions [14]. This demonstrates that even miniaturised *Rhombophryne* species can be distinguished by external morphology from *Stumpffia* species, providing still further support for the recognition of *Rhombophryne*, *Stumpffia*, and *Anilany* as separate genera.

### More than one way to shrink a frog

Paedomorphosis constitutes the retention of characteristics typical of earlier developmental stages of an ancestor in later stages of development [54–56]. The vast majority of amphibians achieve reduced body size via paedomorphosis, retaining in particular paedomorphic head morphology (relatively larger eyes, larger relative brain-case size) [5–8, 13, 14, 57–62]. The species we have described here of the genera *Mini* and *Anodonthyla* fit this pattern, but *Rhombophryne proportionalis* demonstrates that this is not the only way that anurans can achieve reduced body size.

*Rhombophryne proportionalis* would probably have been considered by Alberch et al. [55] and Gould [54] to represent a case of ‘proportional dwarfism’, that is, a reduction in body size without alteration of body proportions, relative to the ancestral shape. Klingenberg [56] considered cases of proportional dwarfism sufficiently rare that he more or less dismissed them and considered it impossible for body size to reduce without resulting in related changes in shape. Yet, while the proportions of *R*. *proportionalis* do not perfectly match those of larger *Rhombophryne* species, it certainly is nearer to their proportions than any other cophyline frog of comparable size (compare [14], and note its short and rounded snout in Fig 2) and its skull morphology in particular is almost unmodified compared to other *Rhombophryne* species (e.g. compare Fig 11 with *R*. *serratopalpebrosa* species group [25]), except in the anterior shift of the jaw joint, which is common in miniaturised frogs [62]. Its proportions differ from those of juvenile *Rhombophryne*, for example in having a proportionally shorter head (HL/SVL 0.20–0.24 vs 0.31 and 0.33 in juvenile *R*. *ornata* and *R*. *regalis*, respectively; unpublished data). It is also the smallest of the cophylines to retain vomerine teeth, and these again are not like those of juvenile *Rhombophryne* (which are disproportionally large) but rather more like those of adult, large-bodied species. Thus, *R*. *proportionalis* is a remarkable case of miniaturisation through proportional dwarfism.

Loss of digits, while a corollary of miniaturisation, is not a form of paedomorphosis [63], but rather a commonly arising morphological homoplasy resulting from miniaturisation through functional constraint of the limb primordium [2, 63, 64]. In our recent revision of the genus *Stumpffia*, we commented on the pattern of digit loss across that genus [14], and noted that it apparently departed from known patterns of digit reduction in reduced frogs [62, 63]. *Mini* has reduced its digits in a manner comparable with *Stumpffia*, and its reduction is further accentuated by the apparent loss of carpal 2. Curiously, although *M*. *mum* is smaller than almost all *Stumpffia* species, its toes are less reduced than the smallest *Stumpffia*, suggesting that absolute adult body size is not the only factor determining the degree of digital reduction; data on the embryos of extremely miniaturised cophylines are needed to understand if limb bud size may be responsible for this change.

The digits of *Anodonthyla eximia* retain the hallmarks of *Anodonthyla*, most obviously in the long cultriform prepollex that characterises males of that genus, but also in the possession of a T-shaped phalanx on the third finger. Its digital formula is also only weakly reduced. In this context, it is, once again, remarkable that *R*. *proportionalis* has undergone only minimal digital loss, having reduced the first phalanx of the first toe, and has not undergone any loss of the fingers, which sets it apart from comparatively miniaturised cophylines. More notably still, its relative finger lengths (see Fig 11) are highly similar to those of larger *Rhombophryne* (e.g. [25]). Both of these cases suggest that miniaturisation is occurring in a contingent, rather than a deterministic fashion in these frogs.

### Underestimated diversity of miniaturised frogs

Globally, miniaturised frogs are thought to comprise a unique ecomorph, specialised to hide in small places and occupy a lower position in the trophic ladder than larger species [5]. Part of the reason this ecomorph has only recently been acknowledged [6] is taxonomic underestimation; it was not possible to understand how many lineages have evolved independently to occupy the miniaturised frog niche while so much of the diversity was undescribed. The diversity was also further obscured by morphology-based supraspecific taxonomy influenced by homoplastic characters [2] that suggested fewer instances of miniaturisation than revealed by DNA-based phylogenetics. In light of the recent taxonomic descriptions (e.g. [5, 6, 10, 14, 57, 60, 65–69]) and strongly supported phylogenetic studies of miniaturised frogs however (e.g. [8, 14, 58, 65, 70–74]), it is becoming clear that the diversity of these frogs is astonishingly high, at both the species and supraspecific levels. The emerging picture of the diversity of miniaturised lineages of cophyline microhylids is a perfect example: newly discovered small microhylids from Madagascar were generally assigned ad hoc to *Stumpffia* based on their size, although keys for described species did exist [75, 76]. Genetic differentiation of different undescribed lineages initially assigned to *Stumpffia* flagged them as belonging to separate deep clades, prompting closer inspection that has yielded the new species described here. This transition of understanding drives home the importance of genetics in shedding light on groups with potentially extensive ecology-linked homoplasy, like fossorial squamates (e.g. [77, 78]).

Based on current understanding of the phylogeny of the Cophylinae [15, 20], the five new species of miniaturised frogs described here represent three additional separate cases of extreme miniaturisation of body size. The total evolutionary lability of cophyline body size is thus only just emerging: *Stumpffia* and *Anilany* have also miniaturised, but due to instability of their relationships in available phylogenetic hypotheses with respect to *Rhombophryne*, we cannot currently establish if they reduced in size independently or if they stem from a small or miniaturised common ancestor. *Platypelis karenae* (SVL 16.1–18.3 mm) and *P*. *tetra* (17.5–19.4 mm among the type series) are also miniaturised species [79], but uncertainty surrounding their taxonomic position means that body size evolution in *Platypelis* is unclear. Finally, and perhaps most remarkably, two of the largest species of microhylids in the world, *Plethodontohyla inguinalis* and *Platypelis grandis*, are also members of this subfamily. To understand the apparently volatile evolution of body size within cophylines, and indeed other aspects of their evolutionary history such as ecology (see below) and digits (see above), detailed analysis will need to be conducted on a robust and densely sampled phylogeny—a project on which we are currently working. Further future expansions should look at patterns across the whole of the Microhylidae, as the Cophylinae are just one of the several subfamilies that exhibit remarkably extensive, interdigitated miniaturisation [8, 11, 13, 65].

### Drivers of miniaturisation in frogs

Lehr and Coloma [7] suggested that miniaturisation may enable frogs to exploit new food resources that are not available to larger frogs. Kraus [5] expanded on this hypothesis, suggesting that miniaturisation may evolve as a means to exploit leaf-litter or mossy habitats; doing so may simultaneously open access to prey that is inaccessible to larger species that cannot penetrate these complex habitats, while also providing enhanced shelter from predators. But exploiting this opportunity comes at the cost of a higher surface-area–volume ratio, leading to more rapid desiccation [80], which Kraus [5] considered as an explanation for the distribution of the smallest frogs exclusively in wet, tropical forests.

Our current data, especially with regard to the new species described here, appears to corroborate these hypotheses. Almost all of Madagascar’s miniaturised microhylids are leaf-litter dwellers, except some few *Stumpffia* species from high elevations (e.g. *S*. *tridactyla*) that are also associated with moss. *Anodonthyla eximia* in particular seems to support the hypothesis of Kraus [5] that miniaturisation is an evolutionary means of exploiting leaf-litter and mossy habitats: In general, *Anodonthyla* are arboreal or scansorial frogs [24, 41]. *Anodonthyla eximia* is not only considerably smaller than any other member of the genus (11.3 mm SVL; the next smallest species is *A*. *pollicaris* at 16.3–18.3 mm SVL) but is also the only apparently terrestrial, leaf-litter-dwelling member of the genus. The transition to miniaturisation in this species thus seems to have come hand-in-hand with a transition to terrestriality. Complementing the very rudimentary natural history information available for this enigmatic species should be a high priority for future field studies in Ranomafana National Park.

Curiously, the miniaturised and arboreal species *Platypelis karenae* (mentioned above) seems to contradict this prediction. Perhaps leaf-axil phytotelmic habits of this species have allowed it to miniaturise without ecological transition by exploiting smaller leaf axils than are available to larger frogs—a hypothesis compatible with our own observations, and, for example, *Microhyla borneensis*, an extremely miniaturised microhylid that breeds in pitcher plants [10, 11]. As a further deviation, the pyxicephalid *Microbatrachella capensis* (15–18 mm SVL) occurs in the South African fynbos shrubland vegetation, with acidic water and a dense humus layer, and breeds in swamps where its regular exotrophic tadpoles develop, while other miniaturized species of *Cacosternum*, such as *C*. *boettgeri*, (18–19 mm SVL) even occur in drier grassland areas, without apparent dependence on large quantities of leaf litter [81]. These cases indicate potential for uncoupling of typical trends of ecological change that accompany miniaturisation, similar to the pattern observed in African puddle frogs, Phrynobatrachidae, wherein terrestrialisation from predominantly aquatic habits was found to be uncoupled from miniaturisation [70]. Studying these exceptional cases in greater detail may reveal additional factors involved in miniaturisation, and drivers of miniaturisation without terrestrialisation.

## Supporting information

S1 Table GenBank accession numbers for 3’ *16S rRNA* sequences used in this study to calculate uncorrected pairwise distances. Numbers in red are those newly produced in this study.
(XLSX)
